# How soluble misfolded proteins bypass chaperones at the molecular level

**DOI:** 10.1038/s41467-023-38962-z

**Published:** 2023-06-21

**Authors:** Ritaban Halder, Daniel A. Nissley, Ian Sitarik, Yang Jiang, Yiyun Rao, Quyen V. Vu, Mai Suan Li, Justin Pritchard, Edward P. O’Brien

**Affiliations:** 1 Department of Chemistry, Pennsylvania State University, University Park, PA 16802 USA; 2 Molecular, Cellular and Integrative Biosciences Program, The Huck Institutes of the Life Sciences, Pennsylvania State University, University Park, PA 16802 USA; 3 Institute of Physics, Polish Academy of Sciences; Al. Lotnikow 32/46, 02-668 Warsaw, Poland; 4 Institute for Computational Sciences and Technology; Quang Trung Software City, Tan Chanh Hiep Ward, District 12, Ho Chi Minh City, Vietnam; 5 Department of Biomedical Engineering, Pennsylvania State University, State College, PA 16802 USA; 6 Huck Institute for the Life Sciences, Pennsylvania State University, State College, PA 16802 USA; 7 Bioinformatics and Genomics Graduate Program, The Huck Institutes of the Life Sciences, Pennsylvania State University, University Park, PA 16802 USA; 8 Institute for Computational and Data Sciences, Pennsylvania State University, University Park, PA 16802 USA; 9 Present Address: Department of Statistics, University of Oxford, Oxford, OX1 3LB UK

**Keywords:** Chaperones, Chaperones, Computational biophysics

## Abstract

Subpopulations of soluble, misfolded proteins can bypass chaperones within cells. The extent of this phenomenon and how it happens at the molecular level are unknown. Through a meta-analysis of the experimental literature we find that in all quantitative protein refolding studies there is always a subpopulation of soluble but misfolded protein that does not fold in the presence of one or more chaperones, and can take days or longer to do so. Thus, some misfolded subpopulations commonly bypass chaperones. Using multi-scale simulation models we observe that the misfolded structures that bypass various chaperones can do so because their structures are highly native like, leading to a situation where chaperones do not distinguish between the folded and near-native-misfolded states. More broadly, these results provide a mechanism by which long-time scale changes in protein structure and function can persist in cells because some misfolded states can bypass components of the proteostasis machinery.

## Abstract (web summary)

How soluble misfolded proteins can bypass chaperones is unknown. Utilizing a meta-analysis, multi-scale modelling, and new experimental data it is found that this phenomena is common and arises from misfolded states that are native-like and long-lived due to protein self-entanglements.

## Introduction

Some soluble, misfolded proteins can bypass the refolding action of chaperones in vivo according to folding and functional assays^1–3^. Typically, in these assays the protein of interest is purified after it has been expressed either heterologously or constitutively from different synonymous messenger RNA (mRNA) variants. A synonymous mRNA variant is an mRNA molecule where one or more codons have been replaced by a synonymous codon, which does not alter the encoded protein’s primary structure but alters the mRNA’s nucleotide sequence.

For example, introducing synonymous mutations into the Chloramphenicol acetyltransferase (CATIII) enzyme decreased its specific activity by 20%^4^. Since the specific activity is an ensemble average over the soluble fraction of proteins, it can be inferred that these synonymous mutations caused a portion of the soluble protein molecules to shift into a misfolded ensemble with decreased activity. Many other examples of this phenomenon exist. The ability of soluble FREQUENCY (FRQ) protein to bind to its partner protein ‘White Collar-2’ (WC-2) was decreased by half when a synonymous variant of FRQ was produced^1^. Since FRQ was expressed in vivo, this indicates that chaperones sometimes fail to catalyze the proper folding of soluble, misfolded FRQ protein molecules.

In many of these studies, alternative explanations for the formation of soluble misfolded proteins have been ruled out. Most of these studies have characterized the properties only of soluble protein through the use of centrifugation, ruling out influences from insoluble aggregates. Many also controlled for changing expression levels, ruling out the possibility that it is changing in protein levels causing this phenomenon. Gel assays ruled out the presence of truncated protein forms in a number of studies. Finally, in some studies, native gels, analytical ultracentrifugation, or size-exclusion chromatography were run to rule out the presence of higher-order, non-native oligomers. Complete details of which controls were performed for each of a set of twenty experimental studies are provided in Supplementary Data 1.

Three fundamental questions arise from these observations: How common is it for soluble, misfolded proteins to bypass chaperones? How long does it take for these misfolded states to fold? And, finally, how do some misfolded proteins avoid the refolding action of chaperones at the molecular level? These are biologically important questions because the answer to the first two questions could impact our understanding of how protein homeostasis is maintained in cells. The answer to the final question would help to explain how synonymous mutations can have long term impacts on protein structure and function in vivo.

**Fig. 1:**
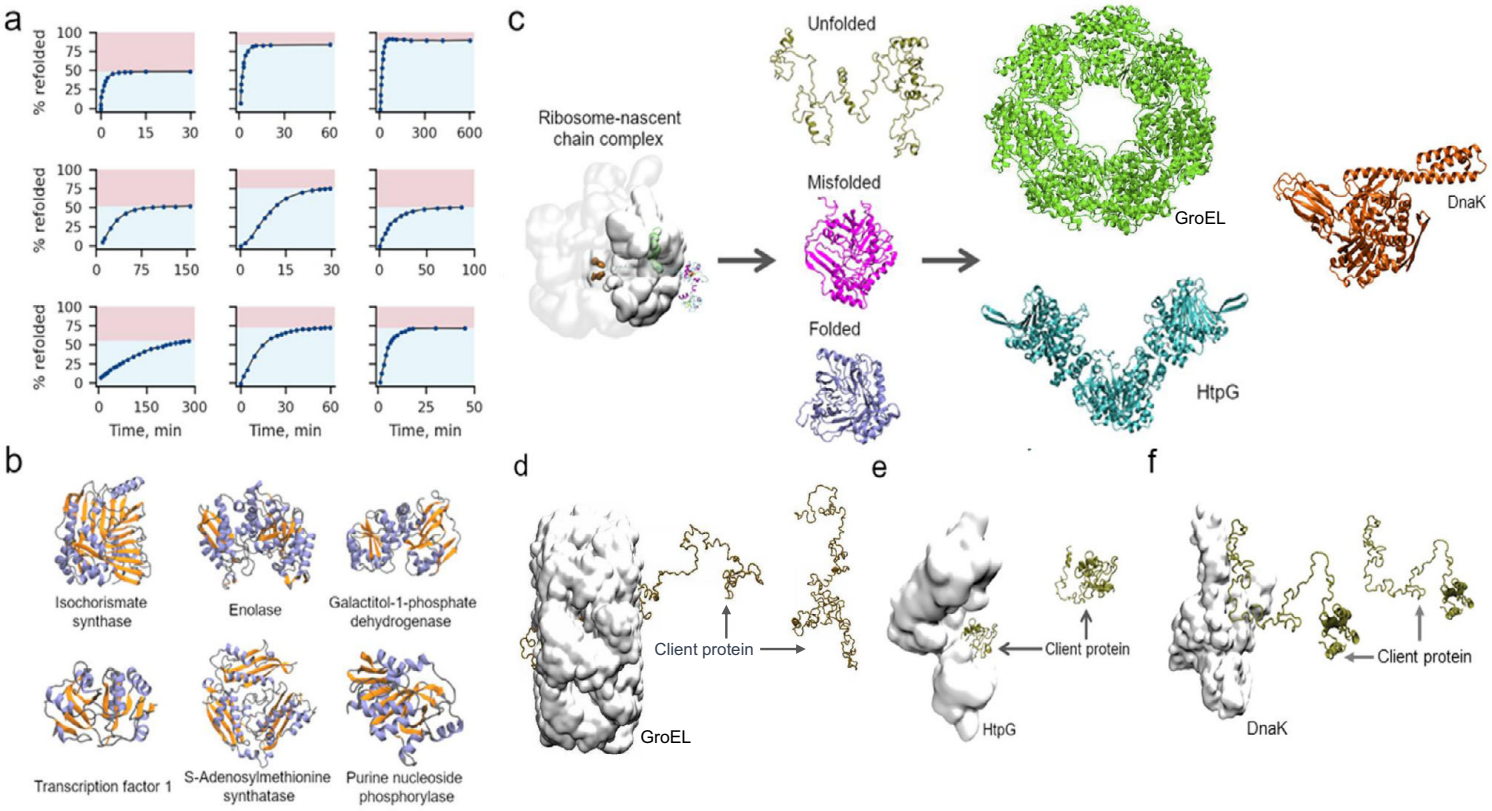
Meta-analysis of protein refolding studies and representations of GroEL, DnaK, HtpG, and client proteins. **a** Through a meta-analysis of the experimental literature we find an appreciable fraction (indicated by the red shaded region of each subplot) of protein molecules bypass chaperones in vitro even though they are not folded (i.e., have not regained activity), and take a minimum of days or longer to reach their folded functional state. **b** Cartoon models of the native state reference structures for six proteins whose interactions with GroEL/DnaK/HtpG we model. Helix, sheet, and loop regions are colored light purple, orange, and grey, respectively. **c** Unfolded, misfolded, and folded conformations were generated by synthesizing each protein using a coarse-grain ribosome-nascent chain complex. After ejection from the ribosome the nascent protein may remain unfolded, reach a misfolded state, or fold. These conformational states may then interact with several post-translational chaperones such as GroEL, DnaK and HtpG. **d** Characteristic structures in both the bound and unbound states of GroEL (white space-filling model) and Isochorismate synthase (brown cartoon). **e** Representative structures in both bound and unbound states of HtpG and Purine nucleoside phosphorylase and **f** Representative structures in both bound and unbound states of DnaK and Purine nucleoside phosphorylase.

To address these questions, we carried out a meta-analysis of the experimental literature focused specifically on in vitro studies where quantitative measurements can be carried out with appropriate controls (Fig. 1a and Supplementary Data 1). We find that subpopulations of soluble, misfolded proteins unaffected by the presence of chaperones are the norm rather than the exception and estimate that in the absence of side reactions, these misfolded states likely take days or longer to fold. To answer the third question, we use coarse-grained and all-atom molecular dynamics to simulate the interactions of newly synthesized proteins with the post-translational chaperones GroEL, DnaK, and HtpG (Fig. 1b–f) and identify how some misfolded states can energetically and structurally bypass these chaperones.

## Results

### Soluble misfolded proteins bypass the *E. coli* chaperone machinery in vitro

We carried out a meta-analysis of the experimental literature reporting time-courses of protein refolding and acquisition of function (Fig. 1a and Supplementary Data 1). We focus on in vitro studies because they are capable of controlling for a number of factors that are currently not possible to control for in vivo. A typical experiment involves splitting a purified protein sample into two test tubes, applying a denaturant (such as urea) to one sample, then performing a dilution jump experiment to initiate protein refolding and measuring the time course of the fraction of functional protein. For such a study to make it into our analysis we require: (i) that the signal be normalized by the activity of the non-denatured protein sample; (ii) that centrifugation be performed before the start of the experiment to remove insoluble aggregates; and (iii) that the fraction of native/functional protein be measured in the presence of one or more chaperones.

Twenty papers spanning three decades meet these criteria^5–24^ (see Table 1 and Supplementary Data 1). Five different chaperones are represented in these studies – GroEL (HSP60), DnaK (HSP70), HtpG (HSP90), HSP33 and HdeA – and nine different client proteins – Malate dehydrogenase, Rhodanese, Luciferase, Rubisco, Aconitase, Peptidase Q, Interferon gamma, Dihydropicolinate synthase, and Galactosidase. Eighteen of these studies measured protein folding in the presence of one chaperone, and two studies used two different chaperones. The duration of the time-courses monitoring refolding in these studies ranged from 30 minutes to 600 minutes, with an average of 150 minutes and a median of 140 minutes. Details are summarized for each study in Table 1, extensive details are reported in Supplementary Data 1, and time courses reproduced in Supplementary Fig. 1. Standard chemical or thermal denaturation procedures are followed in these studies that are unlikely to cause chemical damage to these proteins during the time course of the experiments. Some experiments were performed in triplicate suggesting random experimental errors, such as sticking of proteins to the plastic tips, tube walls or cuvette walls, should average out.

**Table 1:** Meta-analysis of proteins that remain soluble and misfolded in the presence of chaperones using Eq. 1. See also Supplementary Table 1

Protein name	Chaperone(s)	%Misfolded and soluble (a_1_ in Eq. 1)	Slow folding time constant, (min)	k_2_ (min^−1^)	k_2_, Upper bound	k_2_, Lower bound	Reference number
Aconitase	GroEL	57	10^3^	2.18 × 10^−3^	2.22 × 10^−3^	2.14 × 10^−3^	5
Peptidase Q*	GroEL	19	10^4^	1.65 × 10^−4^	0.36 × 10^−2^	1.9 × 10^−23^	6
Luciferase*	HSP70/DnaK	8	10^22^	9.34 × 10^−22^	2.55 × 10^−14^	4.67 × 10^−22^	7
Luciferase	HSP33	43	10^16^	1.71 × 10^−16^	1.72 × 10^−16^	1.39 × 10^−33^	8
Rhodanese	GroEL, Dnak, GrpE	26	10^16^	1.80 × 10^−16^	3.74 × 10^−20^	1.21 × 10^−16^	9
Malate dehydrogenase*	Hdea	50	10^18^	4.90 × 10^−18^	4.62 × 10^−4^	4.54 × 10^−22^	10
Luciferase	HSP70, HSP90	35	10^2^	1.50 × 10^−2^	1.58 × 10^−2^	1.43 × 10^−2^	11
Rhodanese*	HSP60	26	10^19^	1.06 × 10^−19^	2.21 × 10^−19^	5.31 × 10^−20^	12
Malate dehydrogenase*	cpn60	27	10^23^	5.62 × 10^−23^	1.11 × 10^−3^	2.81 × 10^−23^	13
Rhodanese*	GroEL	17	10^3^	9.80 × 10^−3^	4.40 × 10^−2^	2.06 × 10^−20^	14
Rubisco^†^	GroEL	38	10^2^	7.80 × 10^−2^	8.18 × 10^−2^	7.44 × 10^−2^	15
Rubisco	GroEL	70	10^20^	4.82 × 10^−20^	2.85 × 10^−14^	7.27 × 10^−19^	16
Luciferase	HSP70, HSP90	40	10^19^	9.85 × 10^−19^	4.58 × 10^−16^	6.46 × 10^−19^	17
Rubisco*	GroEL	10	10^12^	1.01 × 10^−12^	9.72 × 10^−3^	5.95 × 10^−26^	18
Rubisco	GroEL	23	10^23^	2.07 × 10^−23^	3.58 × 10^−13^	1.03. × 10^−23^	19
Interferon	GroEL	25	10^3^	7.23 × 10^−3^	7.71 × 10^−3^	6.80 × 10^−3^	20
Galactosidase	HSP70/DnaK	43	10^22^	2.19 × 10^−22^	7.75 × 10^−15^	1.09 × 10^−22^	21
Rubisco	GroEL	14	10^14^	3.14 × 10^−14^	4.73 × 10^−13^	1.90 × 10^−25^	22
Malate dehydrogenase	GroEL	6	10^13^	1.39 × 10^−13^	3.19 × 10^−13^	6.96 × 10^−21^	23
Rhodanese	GroEL	20	10^20^	9.34 × 10^−16^	2.92 × 10^−20^	1.97 × 10^−20^	24

^†^This rate constant has units of h^−1^ rather than min^−1^.

^*^These upper and lower bounds on the slowest rate constant were obtained using the error bars reported in the original study. If the original study did not report error bars, we assumed 1% uncertainties on the original data points.

**Fig. 2:**
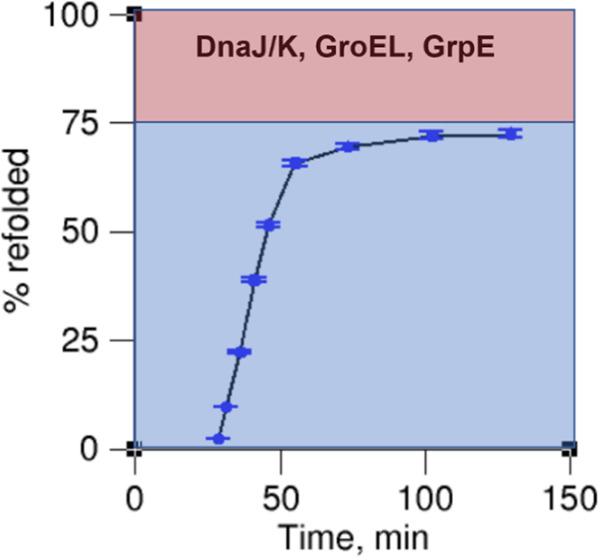
An example time course of reactivation/refolding of soluble rhodanese in a mixture of the chaperones DnaJ/K, GroEL, and GrpE. Rhodanese was initially unfolded using guanidine hydrochloride and refolding then monitored after a dilution jump and the addition of chaperones. Note that >25% of Rhodanase (red shaded region) is unable to reach its fully folded conformation even in the presence of these chaperones during the time course of the experiment. A kinetic fit (Eq. 1) indicates this subpopulation will take 10^16 ^min (2.35 × 10^14^ years, 95% Confidence Interval [2.24 × 10^14^ years, 6.94 × 10^14^ years] assuming 1% error in the measurements) to fold. Experimental data were extracted from ref. ^9^ Fig. 4a using PlotDigitizer (see Supplementary Data 1).

In all of these studies, there is always a fraction of soluble protein that does not attain a folded, functional state by the last time point. The percentage of molecules that did not fold ranged from a low of 6% to a high of 70% (Table 1, Fig. 1a and Supplementary Data 1). Since structure equals function, these percentages indicate there is an appreciable fraction of protein molecules that are soluble, misfolded, and kinetically trapped in solution. Thus, there is always a subpopulation of soluble proteins that misfolds and whose folding is not catalysed by the presence of these chaperones. One example is shown in Fig. 2, where the unfolded client protein Rhodanese is incubated with GroEL/GroES, DnaK, and co-chaperones GrpE and DnaJ. In this example, even 150 minutes after refolding was initiated with a dilution jump, a little more than 25% of soluble Rhodanese remains misfolded.

### Refolding of soluble, misfolded states take days or longer

The folding time courses reported in the literature allow us to roughly estimate how long it takes for the subpopulation of soluble, misfolded states to fold and function. Applying to the experimental time courses a double exponential fit, representing folding to the native state through two parallel pathways, one fast and one slow (see Methods, Supplementary Fig. 1, Supplementary Note 1 and Supplementary Table 1), and interpreting the slower characteristic time scale as the folding time of the soluble misfolded fraction, we estimate that these states typically take days or longer to properly fold. (The very slow characteristic time scales beyond 24 h, reported in Supplementary Table 1, have large unquantifiable errors, and should only be interpreted as indicating folding takes several days or more to occur. See also Methods Section.) Thus, we conclude that these soluble misfolded states convert to the native state very slowly on biological time scales.

### Depletion of ATP by GroEL does not explain the lack of refolding

Many chaperones, including GroEL/GroES, require ATP during their catalytic cycles. It is possible then that the 6–70% of molecules that we conclude are trapped in soluble misfolded states in our meta-analysis are the result of inactive chaperones due to ATP depletion during the experiments. To test this, we solved the time-dependent Master Equations for a simplified model of ATP-dependent protein refolding by GroEL (see Methods). This model considers GroEL-catalyzed folding under several assumptions including only the unfolded state of the client protein binds GroEL. Using this simplified model of GroEL-dependent folding, we re-fit the experimental data for the nine data sets involving GroEL (Supplementary Fig. 2) and computed the time-dependent probability of being in a non-native state, ${P}_{{{{{{\rm{NN}}}}}}}^{{{{{{\rm{sim}}}}}}}(t)$. We find, in general, excellent agreement between the kinetic model and experimental data, with Pearson ${R}^{2}$ in the range of 0.99 (Supplementary Fig. 2) for all but the two poorest fits. These poor fits, for the experiments from Refs. ^5^ and ^20^ are likely due to two factors: (*i*) we do not consider reverse transitions from the misfolded and folded states to the unfolded state and (*ii*) we use the same estimated rate parameters for all proteins based on global averages since protein-specific rate information is not available.

Having verified the model, we next predicted the concentration of ATP as a function of time for each of the nine experiments given the reported initial concentrations (Supplementary Table 2). In each study, the initial concentration of ATP is ≥1000 μM, and our model indicates that GroEL/GroES utilizes between 50–300 μM of ATP during the experiments (Supplementary Table 2 and Supplementary Fig. 2). Therefore, there is a large pool of free ATP available at the final time point. These results are consistent with experiments that measured ATP consumption by GroEL of around 50 μM, indicating that ample ATP remains after the time course is completed (see Fig. 5b in ref. ^24^). We conclude that ATP depletion leading to chaperone inactivity does not contribute to the lack of refolding observed in the original experimental data.

### GroEL decreases the amount of misfolding

GroEL promotes protein folding^6,25^. Therefore, our kinetic model of GroEL should reflect this in the fit parameters ${\varphi }_{F}$ and ${\varphi }_{M}$ (Eq. 7 and Supplementary Fig. 2) that correspond, respectively, to the fraction of client protein molecules that partition either into the folded or misfolded state each GroEL cycle. Comparing these to the same quantities in the absence of GroEL we find that ${\varphi }_{M}\left({Bulk}\right)$ is always greater than ${\varphi }_{M}$ (Supplementary Table 3, Eq. 7). This means that folding yield is enhanced and misfolding reduced in the presence of GroEL. This result is not surprising given GroEL’s well established foldase activity, but it serves to illustrate the model yields sensible results, and also allows quantification of the partitioning into these misfolded states.

### Selection of GroEL client proteins that populate long-lived misfolded states

**Fig. 3:**
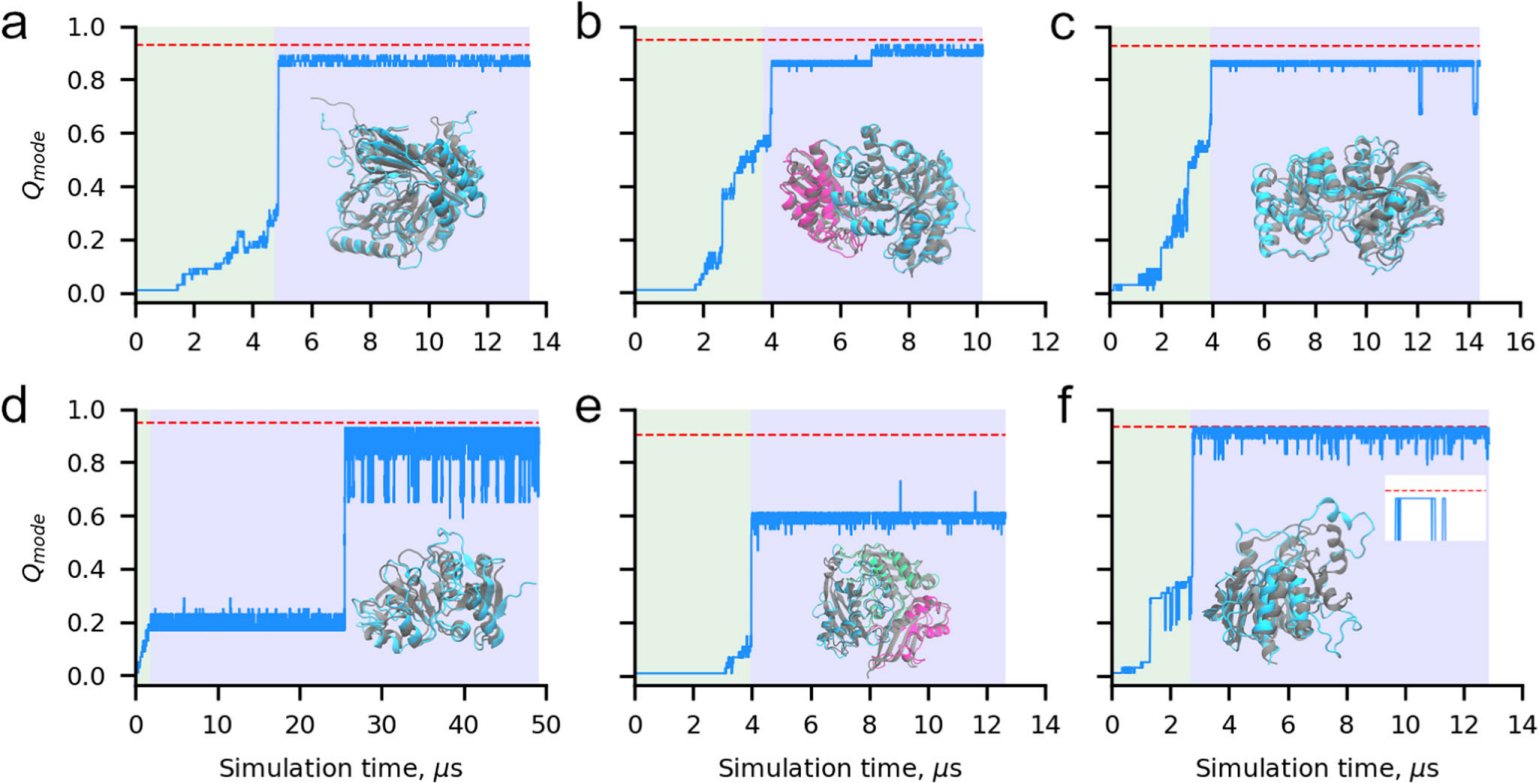
Long-lived misfolded states of six *E. coli* proteins used in the chaperone binding simulations. **a** Time series of ${Q}_{{{{{{\rm{mode}}}}}}}$ for a misfolded trajectory of isochorismate synthase during coarse-grain simulation of protein synthesis (green shaded region), nascent protein ejection from the ribosome (too narrow to view on plot), and post-translational dynamics (purple shaded region). The red dashed line indicates the value of 〈${Q}_{{{{{{\rm{mode}}}}}}}^{{{{{{\rm{NS}}}}}}}$〉, the mean ${Q}_{{{{{{\rm{mode}}}}}}}$ of the native state ensemble (see Methods). A structural alignment of the long-lived misfolded state at the end of post-translational dynamics (cyan) with the native state (grey) is shown as an inset, showing these are near-native misfolded states. **b** Same as (**a**) but for the ${Q}_{{{{{{\rm{mode}}}}}}}$ time series of Enolase Domain 2, which persists in a misfolded state. Enolase Domains 1 (residues 1–127) and 2 (residues 128–432) are displayed in magenta and cyan, respectively. **c** Same as (**a**) but for the ${Q}_{{{{{{\rm{mode}}}}}}}$ time series of a misfolded trajectory of galactitol-1-phosphate dehydrogenase. **d** Same as (**a**) but for the ${Q}_{{{{{{\rm{mode}}}}}}}$ time series of a misfolded trajectory of Transcription Factor 1. **e** Same as (**a**) but for the ${Q}_{{{{{{\rm{mode}}}}}}}$ time series of S-adenosylmethionine synthase Domain 3 from a misfolded trajectory. S-adenosylmethionine synthase Domains 1 (residues 1–10 and 137–233), 2 (residues 11–105 and 234–270), and 3 (residues 106–136 and 271–384) are displayed in magenta, green, and cyan, respectively. **f** Same as (**a**) but for the ${Q}_{{{{{{\rm{mode}}}}}}}$ time series of a misfolded trajectory of purine nucleoside phosphorylase. Inset with white background shows zoomed in view of the final 30 ns of the simulation, demonstrating the native state is not reached. The misfolded structures displayed in (**a**)–(**f**) were used as the initial conformations for the chaperone binding simulations involving interactions with long-lived misfolded states.

Misfolded states can either be short-lived or long-lived^26^. Those that quickly equilibrate to their native conformation are unlikely to have a long-term influence on biochemical and cellular behavior. We therefore searched a dataset of simulations of *E. coli* proteins^27,28^ for those that (i) are experimentally known to bind GroEL, and (ii) populate long-lived misfolded conformations (Fig. 3). We identified six proteins, isochorismate synthase (Fig. 3a), enolase (Fig. 3b), galactitol-1-phosphate dehydrogenase (Fig. 3c), transcription factor 1 (Fig. 3d), S-adenosylmethionine synthetase (Fig. 3e), and purine nucleoside phosphorylase (Fig. 3f) that are confirmed GroEL clients and each display long-lived misfolded states based on comparison of a running average of their fraction of native contacts (${Q}_{{{{{{\rm{mode}}}}}}}$, see Methods) to native state simulations (Fig. 3). We can see in the misfolding trajectory shown in Fig. 3f of purine nucleoside phosphorylase, for example, that this molecule obtains almost all of its native contacts, but there are no fluctuations that allow it to reach the average number of native contacts in simulations started from the native state (red line in Fig. 3f). This indicates that in this single molecule trajectory the protein is kinetically trapped in a near native misfolded state. In trajectories that fold, the native state average is obtained. The previous simulations^27^ indicate that between 20% and 94% of the synthesis trajectories of these proteins misfold, and for almost all these proteins entanglements are the predominant cause of misfolding (Supplementary Table 4).

### Misfolded states have similar binding affinities to GroEL, DnaK and HtpG as the native ensemble

We next asked how it is possible that long-lived misfolded proteins are able to bypass the post-translational cellular chaperone machinery. To address this question we used coarse-grained Langevin dynamics to calculate the dissociation constant between the chaperone GroEL and three distinct conformational states of client proteins: folded, unfolded, and the long-lived misfolded state. In addition to GroEL, we also consider the binding of purine nucleoside phosphorylase to the chaperones DnaK and HtpG (Fig. 1c). It has been found that HtpG on its own does not fold proteins but acts downstream with DnaK^29,30^.

**Fig. 4:**
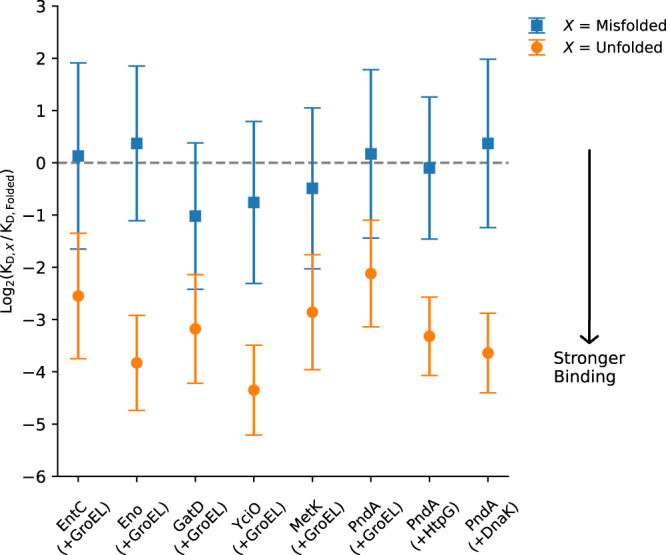
Dissociation constant (log_2_ K_D_) of the unfolded, misfolded, and folded states of client proteins to the chaperones, at 310 K. The folded state K_D_ is used as a reference. Shown for the six client proteins is the log_2_(K_D,*X*_/K_D,Folded_) ratio (where $X$=Misfolded or Unfolded) between the chaperone GroEL and the unfolded ensemble relative to the folded ensemble (U/F, orange circles) and misfolded to folded ensemble (M/F, blue squares). On the x-axis EntC, Isochorismate synthase; Eno, Enolase; GatD, Galactitol-1-phosphate dehydrogenase; YciO, Transcription factor 1; MetK, S-Adenosylmethionine synthetase, PndA, Purine nucleoside phosphorylase, with GroEl ( + GroEL), HtpG ( + HtpG) or DnaK ( + DnaK) present. Error bars represent the 95% confidence intervals about the mean values of 10 independent simulations of each protein conformations. The dotted grey line at log_2_(K_D,*X*_/K_D,Folded_) = 0 occurs when K_D,*X*_ = K_D,Folded_. Differences between orange versus blue points for each protein are statistically significant (maximum p-value is 10^−7^, computed using a two-tailed Permutation Test, see Supplementary Table 6). Negative log_2_(K_D,*X*_/K_D,Folded_) values mean K_D*,X*_ is smaller than K_D,Folded_ (strong binding). Note well, the K_D_’s of the misfolded and folded states are not statistically different, demonstrated by the overlap of the 95% CI with zero and *p* > 0.05.

We find, as expected, that the unfolded ensembles of all six client proteins are more likely to bind to GroEL than their native state ensembles (Fig. 4 and Supplementary Table 5). For all client proteins, the K_D_ values of their unfolded state are always less than the native state value, ranging from 4 to 20-fold smaller than the native state value. For example, transcription factor 1’s unfolded state K_D_ is 20-fold smaller than its native state K_D_, meaning it binds 20 times stronger to the chaperone.

In contrast, the K_D_ values of the folded and misfolded states are statistically indistinguishable, evidenced by the overlap of the 95% confidence intervals in Fig. 4 with zero and the *p*-values > 0.05 reported in Supplementary Table 6. Thus, the misfolded and folded states for all of the proteins have the same or highly similar affinities for interacting with GroEL. We conclude that long-lived misfolded states can bypass GroEL, DnaK, and HtpG because they exhibit no excess interaction with these chaperones beyond that of the native state ensembles’ interaction propensity.

As a quality check, we compared some of these values to literature values. Experimentally, client protein-GroEL K_D_ values have been measured on the order of micro to nanomolar (Supplementary Data 2). Our simulated ${K}_{D}$’s are in this range, having values of 20 to 790 micromolar. Experimentally reported ratios of native to unfolded K_D_’s range from 2- to 30-fold (Supplementary Data 2). Our simulated ${K}_{D}$’s similarly range 4 to 20-fold. Thus, our simulation model is recapitulating realistic ${K}_{D}$ magnitudes and relative differences between two different conformational ensembles, giving more weight to the molecular insights of our model.

As a technical aside, we tested whether our results were arising from finite size effects^31–33^ of the simulation environment. To do this, we reran all the simulations allowing only excluded volume interactions between the client protein and GroEL (Supplementary Table 7), and calculated two sets of odds ratios. First, we calculated the odds ratio (Eq. 4) that the unfolded state and folded state interact with GroEL with and without the attractive term of the Lennard-Jones equation present. In all cases the odds ratio is statistically greater than 1 (Table 2), indicating the primary driving force for unfolded-state binding to GroEL in excess of the folded ensemble is from attractive interactions, not the larger-relative size of the unfolded state in the finite simulation volume (Table 2). Second, we calculated the odds ratio (Eq. 5) of misfolded to folded state binding to GroEL with and without attractive interactions. We find that these ratios are statistically no different than 1, meaning that neither size differences nor interaction differences contribute to differences in native and misfolded state GroEL binding. Thus, the differences in K_D_’s we observe have little influence from finite-size effects.

**Table 2:** Odd’s ratios between probabilities of binding in the unfolded, misfolded, and folded states with and without attractive Lennard-Jones interactions (see Eq. 4 & 5)

Client protein	$\frac{\left(\frac{{P}_{{{{{{\rm{U}}}}}},{{{{{\rm{on}}}}}}}}{{P}_{{{{{{\rm{U}}}}}},{{{{{\rm{off}}}}}}}}\right)}{\left(\frac{{P}_{{{{{{\rm{F}}}}}},{{{{{\rm{on}}}}}}}}{{P}_{{{{{{\rm{F}}}}}},{{{{{\rm{off}}}}}}}}\right)}$	$p$-value^‡^	$\frac{\left(\frac{{P}_{{{{{{\rm{M}}}}}},{{{{{\rm{on}}}}}}}}{{P}_{{{{{{\rm{M}}}}}},{{{{{\rm{off}}}}}}}}\right)}{\left(\frac{{P}_{{{{{{\rm{F}}}}}},{{{{{\rm{on}}}}}}}}{{P}_{{{{{{\rm{F}}}}}},{{{{{\rm{off}}}}}}}}\right)}$	$p$-value
Isochorismate synthase	2.1	8 × 10^−5^	0.75	0.12
Enolase	4.4	1 × 10^−8^	0.84	0.04
Galactitol-1-phosphate-dehydrogenase	3.9	5 × 10^−7^	1.7	0.001
Protein Transcription factor 1	4.5	2.2 × 10^−8^	1.2	0.10
S-adenosylmethionine synthetase	2.8	1.3 × 10^−6^	1.1	0.49
Purine nucleoside phosphorylase	2.4	1.1 × 10^−7^	1.2	0.10

^‡^$p$-values were calculated using a two-tailed Student’s t-test with $\alpha=0.05$.

### A spectrum of chaperone binding affinities for different conformational states

We have only considered three conformational states of proteins. However, proteins populate an ensemble of conformations in solution. Therefore, we expect a range of K_D_ values for different configurational states of the client protein. To estimate this range of K_D_’s, we generated a set of representative conformations by first simulating the heat denaturation of isochorismate synthase and then temperature quenching to 310 K to initiate refolding (see Methods). Next, we sampled 20 different conformations across a range of $Q$ and ${R}_{g}$ (radius of gyration) values that were sampled in these refolding simulations (Supplementary Fig. 3). Finally, we calculated the K_D_ value for each of these conformations interacting with GroEL and plotted them as a function of $Q$ (Supplementary Fig. 3b).

We observe a range of K_D_ values in Supplementary Fig. 3b, from 50 to 600 µM, with a trend of increasing K_D_ with an increasing fraction of native contacts. Below $Q$ < 0.4 (i.e., less structured) entangled states are uncommon, and these less structured states exhibit stronger binding to GroEL. Above $Q$ > 0.4 (i.e., more structured) misfolded entanglements are more common and some have a K_D_ similar to that of the native state (compare, for example, the values of red data points (entangled) around $Q$~0.8 and black circles (folded) at $Q$~0.9 in Supplementary Fig. 3b). Other entangled states have values different than the native state. Thus, we conclude that non-native and misfolded states can have a range of K_D_ values, some similar to the native state, but many not. The greatest predictor appears to be how native-like the non-native state is. Next, we selected nine different conformational states with $Q$ ranging from 0.5 to 0.95 amongst those initial set of twenty structures such that four of them are entangled and performed unrestrained simulations at 310 K. We find that all four entangled states are not able to fold, remaining kinetically trapped throughout the course of the simulation (Supplementary Table 8). On the other hand, the five states with no entanglement all reach the native state during these simulations. These results indicate our model realistically predicts a range of ${K}_{D}$ values depending on how folded the protein is. Further, the slow refolding of entangled states highlights why they are more likely to be biologically relevant than fast-folding non-native states.

### Conclusions are robust to changes in model resolution, binding definition, and initial conditions of refolding

**Fig. 5:**
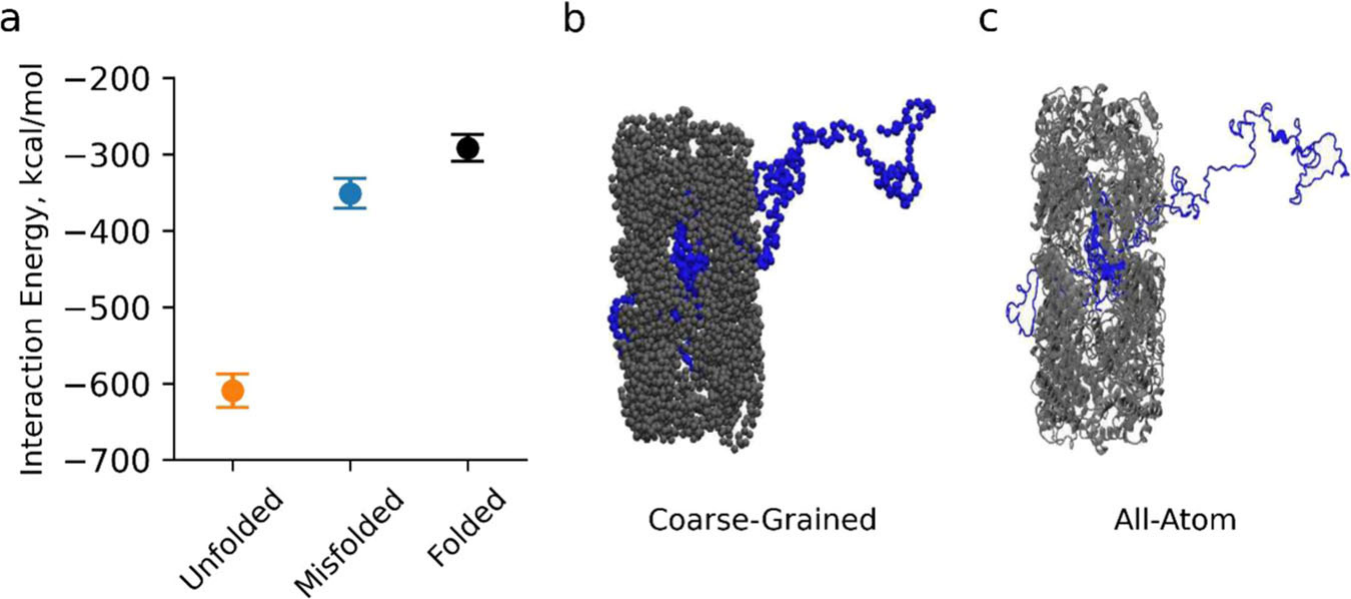
The average interaction energy between Isochorismate synthase and GroEL in an all-atom, fully solvated model. **a** The average all-atom interaction energy between GroEL and unfolded, misfolded, and folded Isochorismate synthase. Each interaction energy is the average from ten independent simulations. Error bars are 95% Confidence Intervals. Even in the higher resolution model, the misfolded state interacts with GroEL similarly to the native state as compared to the unfolded state. Snapshot from the simulations of Isochorismate synthase bound to GroEL in the **b** coarse-grained (before backmapping) and **c** all-atom (after backmapping) representations.

To test if our conclusions are dependent on model resolution we back-mapped each of the ten coarse-grain folded, unfolded, and misfolded conformations of Isochorismate synthase bound to GroEL to an all-atom representation (Fig. 5b and c) and ran 2-ns all-atom simulations in explicit water for each of these 30 systems (see Methods). We then calculated the average interaction energy between Isochorismate synthase and GroEL during the simulations. We find that the interaction energy of the unfolded, misfolded, and folded states are, respectively, −609.5 kcal/mol (95% CI: [−631.0:−588.0]), −351.3 kcal/mol (95% CI: [−370.5:−332.2]), and −291.6 kcal/mol (95% CI: [−308.3:−35.9]) (Fig. 5a). Thus, regardless of model resolution, the misfolded state interaction energies with GroEL are more similar to the folded state than the unfolded state.

We tested whether the contact threshold value (Supplementary Table 9) for defining a chaperone bound state alters our conclusions. To do this we calculate isochorismate synthase’s K_D_ with GroEL as this threshold is varied. We find that our conclusions are unchanged (Supplementary Fig. 4). We also tested whether most of the misfolded states we observe in co- and post-translational folding simulations are seen in temperature quench simulations. For this purpose, we chose the client protein S-adenosylmethionine synthetase and heated it in the computer above its melting temperature and then quenched the temperature to 310 K. We then constructed a log probability plot as a function of two order parameters ($Q$ and $G$, Eq. 6). Comparing these two plots for temperature quench and co/post-translational folding simulations we see that 7 out of the 9 metastable conformational states are the same in both (Supplementary Fig. 5). We note that the misfolded structures used in this study for S-adenosylmethionine synthetase were selected from the highly populated metastable state 2 (Supplementary Fig. 5c, d, yellow) that is present in both ensembles generated by refolding and by synthesis on the ribosome. This demonstrates that most misfolded states are populated in both preparation methods, and the general conclusions of the study are robust.

### Misfolded states bypass chaperones because they are structurally similar to the native state

To understand the structural origins of our binding results we characterized the size, interface, and how native-like each conformational ensemble was by calculating, respectively, the ensemble-averaged radius-of-gyration, solvent accessible surface area, and fraction of native contacts. We observe (Table 3) that the unfolded ensemble is consistently larger and has more exposed surface area than the native state for all client proteins, explaining why it binds more strongly to GroEL, DnaK, and HtpG. In contrast, the misfolded states are much more similar to the native state than they are to the unfolded state. Averaging across all client proteins, the misfolded state is typically 8% larger than the native state (characterized by the percent difference in ${R}_{{{{{{\rm{g}}}}}}}$), has 90% of the native contacts formed, and has a surface area that is only 14% larger than the native state on average. Thus, the misfolded states have structural properties that are similar to the native state, explaining why they interact with these chaperones to a similar degree as the native state.

**Table 3:** Structural characteristics of unfolded, folded, and near-native misfolded states

Client protein	Conformational State	Fraction of native contacts, $Q$	Hydrophobic solvent accessible surface area, Å^2^
Isochorismate synthase	Unfolded	0.50	87.4
	Misfolded	0.85	25.7
	Folded	0.92	20.2
Enolase	Unfolded	0.51	81.1
	Misfolded	0.93	20.9
	Folded	0.94	16.8
Galactitol −1-phosphate dehydrogenase	Unfolded	0.51	77.5
	Misfolded	0.86	24.8
	Folded	0.94	20.1
Transcription factor 1	Unfolded	0.19	98.1
	Misfolded	0.85	29.4
	Folded	0.93	25.3
S-Adenosylmethionine synthetase	Unfolded	0.44	69.3
	Misfolded	0.84	30.5
	Folded	0.94	27.9
Purine nucleoside phosphorylase	Unfolded	0.38	90.4
	Misfolded	0.88	30.3
	Folded	0.93	26.6

**Fig. 6:**
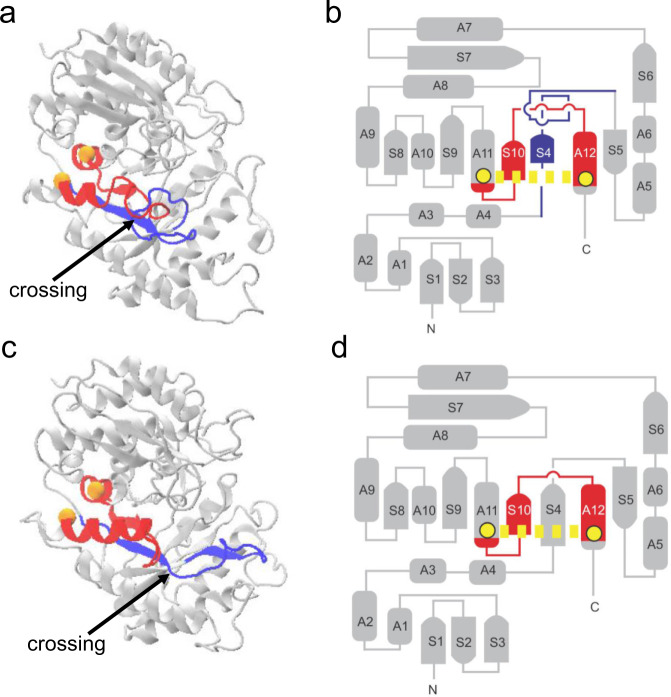
Illustration of Enolase’s near-native, misfolded entangled state and native state. **a** Ribbon representation of the long-lived near-native entangled state of Enolase observed in our coarse-grain simulations. The closed loop and threading segments that form the entanglement are colored red and blue, respectively. The pair of residues that form the native contact that closes the loop are shown as orange spheres at the location of their C_α_ atoms. **b** Flattened secondary structure representation of the misfolded state shown in panel (**a**). In this flattened diagram ‘A’ indicates the location of helices and ‘S’ indicates $\beta$-strands. The yellow spheres represent the pair of residues that forms the native contact (yellow dashed lines) that closes the loop. The threading segment is shown in blue and the closed loop is shown in red. **c** Ribbon representation of the native state of Enolase, which contains no entanglements but for the sake of comparison we color the segments that form the entanglement state the same as in (**a**). **d** Flattened schematic representation of the native state of Enolase with no entanglements. The atomistic structures shown in (**a**) and (**c**) were back-mapped to an all-atom representation from coarse-grain structures for visualization purposes. The structure of Enolase shown in (**a**) is the same misfolded as the structure shown in Fig. 3, panel b but rotated and colored differently.

The reason why these particular misfolded states are kinetically long-lived was previously explained^27,34^. These misfolded states involve non-native changes in non-covalent lasso entanglements. A non-covalent lasso entanglement involves two structural components: a geometrically closed protein backbone loop, and a N- or C-terminal segment that threads through that loop. The loop is closed by a non-covalent native contact. Some 70% of globular proteins contain non-covalent lasso entanglements^35^. A non-native change of entanglement, characterized by our metric $G$ (see Eq. 6 and Methods), means that a protein that forms one of these self-entanglements in the native state does not form it in the misfolded state, while a protein that does not form one of these self-entanglements in the native state does form it in the misfolded state. Each of the six proteins we simulated misfolds into states that exhibit a non-native gain of entanglement relative to the native state. When such non-native changes of entanglement occur in near-native misfolded states, it is an energetically costly and slow process to reach the native state because the protein must unfold to allow the correct entanglement state to be achieved. An illustration of a non-native gain of entanglement (which is present in its misfolded conformation that we simulated) is illustrated for protein Enolase in Fig. 6, where the arrow points to the crossing point of the threading segment through the loop in Fig. 6a. The entanglements in the other five client protein are illustrated in Supplementary Fig. 6 through 10.

### Misfolded states in simulations are consistent with Limited Proteolysis Mass Spectrometry data

We compare the consistency of the misfolded states observed in our simulations with Limited Proteolysis Mass Spectrometry (LiP-MS) data^36^ which reports on specific proteinase K (PK) cut sites in a protein that have changes in peptide abundance upon chemical refolding^37^ (Supplementary Table 10). We can only compare two of the proteins we simulated with the LiP-MS data because the others lack PK cut sites due to low coverage or inconsistent cut sites across time points. We first identify a set of metastable states with conformational and temporal clustering along the Q and G order parameters^34^ followed by a topological analysis of the most probable structures in each metastable state to determine the unique changes in self-entanglement observed in our simulations (Supplementary Tables 11 and 12). We examined the consistency between an entangled conformation and the set of LiP-MS peptides with significant changes in abundance as measured by both primary structure overlap and the consistency of changes in solvent exposure relative to the native state. A permutation test with randomly selected peptides derived from the theoretical distribution of all possible PK cut sites finds that 2-out-of-11 and 10-out-of-19 of these entangled conformations for S-Adenosylmethionine synthetase and Enolase respectfully, are consistent with the experimental data beyond random chance with *p*-values less than 0.05 (Supplementary Table 13). In particular, PK cut sites A60, G317, and Q343 in S-Adenosylmethionine synthetase and P129, M151, M170, G363, and L383 in Enolase have the most statistical significance with the observed misfolded states across all 3 LiP-MS refolding time points (Supplementary Fig. 11). The consistency in the overlap of experimentally observed changes in solvent exposure and our predicted changes in self-entanglement for Enolase is of particular interest in light of the decades-old evidence that it can adopt stable, soluble misfolded conformations^38,39^.

## Discussion

Using a combination of published experimental data, kinetic modeling, and multi-scale simulations we have answered a number of basic molecular biology and biochemistry questions concerning protein structure and function in vivo. The observations that synonymous mutations can have long-term effects on protein structure and function in vivo strongly imply that soluble, misfolded subpopulations persist in cells and that chaperones do not catalyze their folding on biologically relevant time scales. This motivated us to re-analyze the last several decades of literature to examine if there was quantitative in vitro data to test this inference. We indeed find that in every single in vitro experiment in which there are fairly rigorous controls and normalization there is always a subpopulation of soluble, misfolded, less-functional proteins that do not fold in the presence of chaperones. These subpopulations can be as high as 70% of the total protein molecules in solution. Applying a kinetic model to the experimental time courses, we estimate these soluble misfolded states can take a minimum of days or longer to fold in the presence of chaperones. Thus, the in vivo and in vitro data indicate the same phenomenon: some soluble, misfolded proteins can bypass the chaperone machinery for long periods.

These results do not mean that all misfolded and non-native conformations bypass chaperones. At equilibrium, proteins adopt an ensemble of distinct structures with different probabilities, existing on a continuum from more to less ordered and hence, for globular proteins, span from exposing less to more hydrophobic surface area. Thus, some protein conformations will be more or less likely to interact with chaperones, and hence different misfolded conformations will have different affinities for chaperones. Indeed, in our simulation results we observe that when the protein is less ordered and more unfolded the binding affinity for the chaperones increases (Supplementary Fig. 3).

Our kinetic analysis indicates that many of the soluble misfolded states take days or longer to fold. An interesting implication of this is that many proteins will have subpopulations that can be kinetically trapped in soluble metastable states throughout their entire life in a cell as well as over multiple doubling times in E. coli. The median half-life of a protein in exponentially growing E. coli is 241 minutes^40^. Eighteen out of twenty in vitro refolded proteins reported here have a slow folding phase time constant longer than this time. This opens up the possibility of an epigenetic mechanism, where the ‘memory’ of the initial conditions under which a protein folded could be encoded in its structural ensemble and affect cellular properties in subsequent generations.

We ruled out the alternative hypothesis that ATP depletion leads to inactive GroEL resulting in soluble, non-folded proteins in the experiments. Our kinetic model (Supplementary Fig. 12 and Eq. 7) indicates that in each experiment in which GroEL/GroES was present, the ATP concentration remains high throughout the experimental time course. Another hypothesis that can be ruled out is that insoluble protein aggregate formation occurs continuously, preventing attainment of 100% of native state activity. If such protein aggregation occurred during the time course of the experiment more-and-more protein would shift to the non-functional aggregated form leading to a downward slope in the percent refolded versus time (Fig. 1a). Instead, a plateau is observed in the data in all cases, indicating continuous aggregate formation is not occurring.

Our identification of near-native self-entanglements as a mechanism explaining how proteins can remain soluble and misfolded is not mutually exclusive with other misfolding and misfunctioning mechanisms that can occur in vivo. These other mechanisms can include non-native dimer swapped structures^41,42^, aberrant protein isoforms from mRNA splicing^43^, post-translational modifications^44^, and chemical processes that age proteins such as oxidation^45^. Indeed, in our meta-analysis data set, no single study simultaneously ruled out all these possibilities. Most ruled out some, but not all of these confounding factors. Future experiments that seek to detect these non-native changes of entanglement should use a large battery of controls to simultaneously rule out these alternative explanations.

Lasso-like entanglements are common in the native fold of proteins and entanglements more generally have been a subject of interest to the polymer community for 60 years^46–50^. In the 1960’s, scientists noticed entanglements in synthetic polymers^51^, and found polymer composition, polarity, and tacticity can lead to alteration in the frequency and strength of entanglements. And entanglements due to loop threading in proteins have been found to be more common in the case of larger proteins with more than 200 residues^46^.

Another important aspect of our study is that the simulations utilized six proteins that have been previously found^27^ in simulations to populate long-lived misfolded states, and compared their chaperone binding affinity to that of the unfolded and folded states. The fact that these are long-lived misfolded states is biologically relevant for two reasons. First, if the misfolded states rapidly folded they would not need chaperones to acquire their function. Second, it is these kinetically trapped misfolded states that can have long-term impacts on subcellular processes and phenotype through their loss-of-function. Through these comparisons we were able to demonstrate – using both coarse-grained and all-atom protein models – that these misfolded and native states have similar affinities for chaperones, indicating that chaperones do not treat these particular long-lived misfolded states much differently than they do the native state. The structural and energetic origin of this lack of differentiation comes from the high structural and surface similarity of the misfolded and native states. The misfolded states persist for two reasons. They form a non-covalent lasso entanglement – meaning part of their protein backbone created what can be geometrically defined as a closed loop, and the N- or C-terminal segment threads through this loop – but also they contain significant native structure. This combination means that to disentangle and reach the native state large portions of the misfolded protein must unfolded (also known as backtracking^52–55^), which can be a very slow process^56^. Indeed, applying a standard backtracking analysis method^54^, we observe that misfolding protein trajectories must partially unfold (Supplementary Fig. 13a) to reach the native state, whereas fast-folding trajectories exhibit no such behavior (Supplementary Fig. 13b). Hence, the large amount of native structure around an entanglement leads to long-lived states. By choosing to study misfolded states that were kinetically long-lived we concomitantly selected for misfolded states that were native like. Indeed, where possible to compare to experiment, the misfolded structures and LiP-MS data are found to be in excellent agreement.

Backtracking is not unique to coarse-grained structure-based models. It occurs in proteins^52,57,58^, nucleic acids^59^, in models using transferable all-atom force fields^60^ and there is experimental evidence for backtracking in a number of proteins^57,58^. Further, the misfolded entangled states we see in the structural model are also observed in transferable physics-based force fields. Thus, such backtracking occurs in nature and is observed independent of model resolution and forcefield. 33% of globular domains contain *native* non-covalent lasso entanglements^35^. In our simulations, we observe these non-covalent lasso entanglements can occur in misfolded states. Often in nature, if a tertiary structural element can occur in a native state, it has the potential to occur in the misfolded state. Thus, the various components that make up our key conclusions have been seen in various forms in different studies and fields^61^.

Interestingly, two simulation studies^27,34^ predicted that many soluble misfolded states could take anywhere from days to years to fold. Our analysis of the published experimental data indicates these misfolded states take a minimum of days to fold. Thus, the previous simulation predictions are qualitatively consistent with the current results.

It was recently shown that proteins that contain non-covalent lasso entanglements in their native state are more likely to get degraded as newly synthesized proteins, probably because they tend to be slow-folding proteins^62^. This suggests the realistic possibility that when non-covalent lasso entanglements form as off-pathway intermediates, they might have differential rates of degradation as opposed to proteins that do not. Further, because some knotted proteins (another class of entanglement) have been shown to be more resistant to degradation^63^ so too it might be the case that already formed non-covalent lasso entanglements could be more resistant to degradation.

A critique of our meta-analysis is that we only analyzed in vitro data, and the lack of an in vivo environment, which includes vectorial synthesis by the ribosome and the presence of more types of chaperones, artificially increased the subpopulations of soluble misfolded protein. While it is possible that the fraction of soluble, misfolded protein may decrease in the cellular context they are not entirely eliminated. It has been observed, for example, that when a protein is synthesized by the ribosome it still populates states that remain soluble in non-functional form – thus, vectorial synthesis does not eliminate these subpopulations^64^. Additionally, synonymous mutations that alter the speed of translation but not the encoded protein sequence can impact a host of cellular processes^1,65–68^, including a protein’s structure and function in vivo. These observations suggest the molecular explanation from this study is likely to remain relevant in vivo, even if population levels of soluble misfolded states are different compared to in vitro.

A promising connection will be to examine if the type of long-lived misfolded states we observe are relevant to organismal aging. In the case of enolase, there is evidence that it undergoes a thermodynamically reversible conformational change into a kinetically trapped state depending on the age of the organism in which it is expressed^38,39^. Age-related functional changes in aminoacyl-tRNA synthetases have also been suggested to arise from conformational changes in some organisms^69^. A challenge in such studies will be to control for side reactions, such as increased oxidative damage that can occur to non-native conformations compared to their native counterpart^70–73^. Thus, the phenomena we have identified may be of relevance to some of the molecular origins of aging.

These and other recent findings^74^ are providing a new perspective on protein structure and function in vivo suggesting proteins commonly exhibit subpopulations of structural ensembles that are soluble, misfolded, less functional, not rapidly degraded, not quick to aggregate, nor acted upon excessively by chaperones. The population of molecules with these characteristics can be influenced by both translation-elongation kinetics, as suggested by synonymous mutation studies, or through denaturation and renaturation, as seen in our meta-analysis. It is natural to hypothesize other perturbations could influence their populations as well, such as changes in temperature^75^. Experimental efforts to structurally characterize these self-entangled states are likely to be a fruitful area of future research, as the implications of these states for protein structure, function, and homeostasis are broad and fundamental.

## Methods

### Extrapolation of refolding timescales

Raw data were extracted from the published experimental papers listed in Table 1 using PlotDigitizer (http://plotdigitizer.sourceforge.net/). These raw values, which represent the percent refolded as a function of time, were then converted to the percent non-native as a function of time by taking %non-native$=100-$%refolded. The resulting %non-native versus time data series were then divided by 100%, giving the time-dependent probability of the protein being non-native, ${P}_{{{{{{\rm{NN}}}}}}}\left(t\right)$, and then fit with the equation

1 $${P}_{{NN}}\left(t\right)={a}_{0}\exp \left(-{k}_{1}t\right)+{a}_{1}\exp \left(-{k}_{2}t\right)$$

In Eq. 1, $t$ is time, and ${k}_{1}$ and ${k}_{2}$ are refolding rate constants. $t$=0 corresponds to the time at which folding conditions were established. A similar procedure was previously used to extract characteristic slow-folding timescales for protein folding via an obligate misfolded/intermediate state^27^. This kinetic scheme^76^ (Supplementary Note 1) represents processes in which $A\to N$ and $B\to N$ are parallel pathways with no interconversion between ensembles $A$ and $B$. $A$ and $B$ represent the fast- and slow-folding (i.e., misfolded) populations, respectively, and $N$ represents the natively folded protein. The rate constants ${k}_{1}$ and ${k}_{2}$ thus correspond to the rates of folding for the fast- and slow-folding populations, respectively. The values ${a}_{0}$ and ${a}_{1}$ represent, respectively, the initial probability of being in state $A$ and $B$, with ${a}_{0}+{a}_{1}\equiv 1$. Supplementary Fig. 1 displays the experimental data, ${P}_{{{{{{\rm{NN}}}}}}}\left(t\right)$ values, and fit results, while Supplementary Table 1 summarizes all fit parameters. Non-random residuals using a single exponential fit (Supplementary Fig. 14) demonstrate that the double exponential fit (Eq. 1) better describes the data (Supplementary Fig. 15).

${k}_{2}$ rates reported in Supplementary Table 1 below 10^−3^ min^−1^ suffer from large and growing errors the smaller ${k}_{2}$ becomes, and therefore should only be interpreted to indicate that folding is taking a day or longer. To illustrate why this is consider that the longest experimental time course reported is 300 minutes. As ${k}_{2}$ gets smaller the argument $-{k}_{2}t$ in Eq. 1 tends towards zero. Therefore, we can approximate Eq. 1 using a Taylor expansion to the 1^st^ order on the second term, resulting in ${P}_{{NN}}\left(t\right)\approx {a}_{0}\exp \left(-{k}_{1}t\right)+{a}_{1}-{{a}_{1}k}_{2}t$. That is, the decay of the non-native state is a convolution of an exponential decay, a linear decrease whose slope is ${{a}_{1}k}_{2}$, and a constant ${a}_{1}$. To be able to accurately determine ${k}_{2}$ it is required we be able to observe in the experimental time course a linear regime whose slope can be measured. As a general rule of thumb, observing a one-tenth change in slope during the experiment should yield reasonable estimates of ${k}_{2}$. This means that ${k}_{2}$ rates that are 10 times longer than the experimental time course can be measured. Thus, since most of the reported experiments are on the order of 100 minutes, characteristic decay times of 1000 minutes can be measured, and taking the inverse means rates on the order of 10^−3 ^min^−1^ can be estimate. Beyond this, ${k}_{2}t$ becomes so small that ${P}_{{NN}}\left(t\right)$ is better modelled using a zeroth order Taylor expansion, yielding ${P}_{{NN}}\left(t\right)\approx {a}_{0}\exp \left(-{k}_{1}t\right)+{a}_{1}$.

### Selection of chaperones and client proteins

Monomers composing the molecular chaperone GroEL consist of the apical, equatorial, and interconnecting domains. Client proteins bind to a specific region within the apical domain. All structures of GroEL used in this study are based on PDB structure 1KP8, which has been used widely in GroEL simulation studies^77,78^. We model client proteins interacting with one GroEL heptameric ring. We modeled the interactions of six client proteins with GroEL: (1) Transcription factor 1 (PDB ID: 1K7J), (2) purine nucleoside phosphorylase (PDB ID: 1A69), (3) S-adenosylmethionine synthetase (PDB ID: 1P7L), (4) enolase (PDB ID: 2FYM), (5) isochorismate synthase (PDB ID: 3HWO), and (6) galactitol-1-phosphate dehydrogenase (PDB ID: 4A2C). We selected these proteins because they are confirmed GroEL clients^79–81^ and each was previously observed to populate long-lived misfolded states in coarse-grained simulations of protein synthesis, ejection, and post-translational dynamics^27^.

For each of these proteins, we selected structures representing long-lived misfolded conformations from synthesis trajectories based on comparison of their ${Q}_{{{{{{\rm{mode}}}}}}}$ to the average ${Q}_{{{{{{\rm{mode}}}}}}}$ values computed from simulations initiated from the native state coordinates (Fig. 3). The fraction of native contacts, $Q$, was first calculated for each domain and interface of each protein during nascent protein synthesis, ejection, and post-translational dynamics. Only contacts between pairs of amino acids that are both within the set of secondary structural elements identified by STRIDE^82^ in the native-state reference structure are considered. ${Q}_{{{{{{\rm{mode}}}}}}}$ was then computed as the mode of these $Q$ values within a 15-ns sliding window and compared to reference values computed as the average ${Q}_{{{{{{\rm{mode}}}}}}}$ over all windows of ten independent simulations started from the native state, denoted $\langle {Q}_{{{{{{\rm{mode}}}}}}}^{{{{{{\rm{NS}}}}}}}\rangle$. Trajectories are considered to populate long-lived misfolded states if they never reach $\langle {Q}_{{{{{{\rm{mode}}}}}}}^{{{{{{\rm{NS}}}}}}}\rangle$ during the simulation. Trajectories representing long-lived misfolded states for each of the six GroEL client proteins listed above were identified in this way and their final coordinates after post-translational dynamics used as the initial coordinates for the chaperone binding simulations. Structural properties of each of the starting protein conformational states used in these simulations are reported in Table 3. The contact maps of the folded and misfolded conformations of each client protein are shown in Supplementary Fig. 16.

Unfolded conformations for binding simulations were selected for each client protein as the first structure after ejection from the ribosome was complete (75 ps after ejection) in the same trajectories identified to be in long-lived misfolded states. Finally, representative native conformations were chosen for each protein as the final structure from a simulation initiated from the native state coordinates and run for 30 CPU days.

In addition to simulations with GroEL, we also examined the interactions of chaperones DnaK and HtpG with client-protein purine nucleoside phosphorylase (PNP)^83^. Full-length coarse-grained models of DnaK and HtpG were constructed from PDB IDs 5NRO and 2IOQ, respectively. 2IOQ was used in several earlier HtpG simulation studies^84–86^ while the DnaK structure 5NRO was selected because it has been used in earlier studies^87,88^. Simulations of DnaK and HtpG were otherwise conducted in the same fashion as those described for GroEL and its client proteins.

### Construction of coarse-grained protein representations

We use a C_α_ coarse-grained representation for GroEL, DnaK, HtpG, and their client proteins^28,89^. The potential energy $E$ of a conformation is calculated according to the expression

2 $$E=	\mathop{\sum}\limits_{i}{k}_{{{{{{\rm{b}}}}}}}{\left({r}_{i}-{r}_{0}\right)}^{2}+{\sum }_{i}{\sum }_{j}^{4}{k}_{{{{{{\rm{\varphi }}}}}},{ij}}\left(1+{{\cos }}\left[j{\varphi }_{i}-{\delta }_{{ij}}\right]\right) \\ 	+\mathop{\sum}\limits_{i}-\frac{1}{\gamma }{{{{{\rm{ln}}}}}}\left\{{{\exp }}\left[-\gamma \left({k}_{{{{{{\rm{\alpha }}}}}}}{\left({\theta }_{i}-{\theta }_{{{{{{\rm{\alpha }}}}}}}\right)}^{2}+{\varepsilon }_{\alpha }\right)\right]+{{\exp }}\left[-\gamma {k}_{{{{{{\rm{\beta }}}}}}}{\left({\theta }_{i}-{\theta }_{{{{{{\rm{\beta }}}}}}}\right)}^{2}\right]\right\} \\ 	+\mathop{\sum}\limits_{{ij}}\frac{{q}_{i}{q}_{j}{e}^{2}}{4\pi {\varepsilon }_{0}{\varepsilon }_{{{{{{\rm{r}}}}}}}{r}_{{ij}}}{{\exp }}\left[-\frac{{r}_{{ij}}}{{l}_{{{{{{\rm{D}}}}}}}}\right]+\mathop{\sum}\limits_{{ij}\in \left\{{{{{{\rm{NC}}}}}}\right\}}{\epsilon }_{{ij}}^{{{{{{\rm{NC}}}}}}}\left[13{\left(\frac{{\sigma }_{{ij}}}{{r}_{{ij}}}\right)}^{12}-18{\left(\frac{{\sigma }_{{ij}}}{{r}_{{ij}}}\right)}^{10}+4{\left(\frac{{\sigma }_{{ij}}}{{r}_{{ij}}}\right)}^{6}\right] \\ 	+\mathop{\sum}\limits_{{ij}\notin \{{{{{{\rm{NC}}}}}}\}}{\epsilon }_{{ij}}^{{{{{{\rm{NN}}}}}}}\left[13{\left(\frac{{\sigma }_{{ij}}}{{r}_{{ij}}}\right)}^{12}-18{\left(\frac{{\sigma }_{{ij}}}{{r}_{{ij}}}\right)}^{10}+4{\left(\frac{{\sigma }_{{ij}}}{{r}_{{ij}}}\right)}^{6}\right]$$

In Eq. 2 the summations represent, from left to right, contributions from virtual C_α_ − C_α_ bonds, torsion angles, bond angles, electrostatic interactions, Lennard-Jones-like native interactions, and repulsive non-native interactions to the total potential energy ($E$) of a given coarse-grain model configuration. The bond, dihedral, and angle terms have been reported elsewhere^90,91^. Electrostatic interactions are described using Debye−Hückel theory with a Debye length, ${l}_{D}$, of 10 Å and a dielectric constant of 78.5. Interaction sites representing the positively charged amino acids lysine and arginine are assigned $q=+ e$, sites representing glutamic acid and aspartic acid are assigned $q=-e$, and all other interaction sites are taken to have a charge of zero^91^. We compute the contribution from native contacts to $E$ using the 12 − 10 − 6 interaction potential of Karanicolas and Brooks^91^. The value of ${\epsilon }_{{ij}}^{{{{{{\rm{NC}}}}}}}$, the depth of the energy minimum for any particular native contact, is calculated as ${\epsilon }_{{ij}}^{{{{{{\rm{NC}}}}}}}={n}_{{ij}}{\epsilon }_{{{{{{\rm{HB}}}}}}}+\eta {\epsilon }_{{ij}}$. ${\epsilon }_{{{{{{\rm{HB}}}}}}}$ represents the energy contribution from hydrogen bonds, while ${\epsilon }_{{ij}}$ represents the energy contribution from the van der Waals contacts between a pair of residues $i$ and $j$ found to be in contact within the protein all-atom reference structure. ${n}_{{ij}}$ indicates the number of hydrogen bonds formed between a pair of residues $i$ and $j$. The value of ${\epsilon }_{{ij}}$ is initially set using the Betancourt−Thirumalai pairwise potential^92^ and multiplied by a constant $\eta$ to construct a reasonably stable coarse-grain model as described below. The collision diameters, ${\sigma }_{{ij}}$, between all the C_α_ interactions sites involved in native contacts are set equal to the distance between the C_α_ atoms of the corresponding amino acid residues in the crystal structure divided by 2^1/6^. van der Waals interaction energies between pairs of residues that do not share a native contact are instead computed in the final summation. For all the non-native interactions, ${\epsilon }_{{ij}}^{{{{{{\rm{NN}}}}}}}$ is set to be 0.000132 kcal/mol and ${\sigma }_{{ij}}$ is computed as reported previously^91^. The average energy value for the native interaction, ${\epsilon }_{{ij}}^{{{{{{\rm{NC}}}}}}}$, is 0.6675 kcal/mol.

### Selection of ***η*** for chaperone and client protein coarse-grain models

To obtain realistic biomolecular stabilities we scale the ${\epsilon }_{{ij}}$ terms in Eq. 2 by a multiplicative factor $\eta$. Values of $\eta$ for all client proteins were taken from a previous study^28^, with different values used for each domain and interface; we reproduce these values for the client proteins studied here in Supplementary Table 14. These $\eta$ values themselves are based on a previous training set of globular proteins^93^. The selection procedure for these $\eta$ values is described in detail in Ref. ^28^. Briefly, sets of ten 1-µs Langevin dynamics simulations were run in CHARMM^94^ version c35b5 at 310 K with a friction coefficient of 0.050 ps^−1^, a 15-fs integration time step, and the SHAKE^95^ algorithm used to constrain all bond lengths. A particular $\eta$ value was considered suitable if the coarse-grain model had a fraction of native contacts, $Q$, greater than 0.69 for ≥98% of simulation time during each of the ten 1-µs simulations with a particular set of $\eta$ values.

We applied this same procedure to select suitable $\eta$ values for the intra- and inter-domain contacts within the chaperone proteins GroEL, HtpG, and DnaK. We chose to use single values for all native contacts for these proteins, rather than domain- and interface-specific values; the results, ranging from 1.400 to 1.800, are listed in Supplementary Table 15.

The values of $\eta$ for chaperone-client protein interactions were selected as the value for each client protein that resulted in the unfolded state binding to the chaperone in 40–60% of simulation frames during the binding simulations described in the next Methods section. Initial simulations were run with the unfolded state using $\eta$ = {0.100, 0.110, 0.120, 0.140, 0.145, 0.150, 0.153, 0.155, 0.160, 0.200} for client-chaperone interactions; the selected values are recorded in Supplementary Table 16. These simulations were run in OpenMM^96^ v7.4.1 as described below using equivalent parameters to the CHARMM simulations described above. Note that attractions between client proteins and chaperones are non-specific, with each client protein interaction site experiencing the same attractive force to each chaperone interaction site.

### Simulation of GroEL, DnaK, and HtpG interactions with client proteins

Simulations were initialized with the center-of-mass of the GroEL coarse-grain model at the origin of the coordinate system. The client protein of interest was then placed in a random orientation such that the distance between its center-of-mass and the center of the top of GroEL ring was 70 Å, with no van der Waals contacts between them. Spherical harmonic restraints, with a force constant 0.1 kcal/(mol  ×  Å^2^), were placed on all GroEL interaction sites to maintain its conformation and position at the origin throughout the simulation. Root Mean Square Deviation (RMSD) restraints with a force constant of 0.1 kcal/ (mol  ×  Å^2^) were used to maintain the client proteins in their initial conformations. This system was then placed in a flat-bottom spherical restraining potential of radius 160 Å. The sphere center was placed such that the client proteins can interact with the surface, cavity, and sides of the GroEL heptamer but cannot access the back side of the GroEL heptamer that would typically be hidden by the other heptameric ring (GroEL is a double ring system). A 160 Å radius was found to easily accommodate each of the client proteins unfolded states. For each unfolded, folded, and near-native misfolded client protein conformation we ran simulations with ten different initial client protein orientations generated by randomly rotating the starting client protein conformation. Each initial conformation was then simulated for 2.4 µs in the presence of GroEL. Simulations of DnaK or HtpG and their client protein purine nucleoside phosphorylase were carried out in an analogous fashion. For DnaK and HtpG, the chaperone was placed at the origin, and a 200 Å radius sphere also centered on the origin was used with the client protein initially placed in a random orientation 50 Å away. All restraints, force constants, and other simulation parameters were otherwise the same as for the GroEL-client protein simulations. All simulations were performed using OpenMM^96^ with a Langevin thermostat at 310 K, a friction coefficient of 0.050 ps^−1^, a 15-fs integration time step, and all bonds constrained.

### Calculation of K_D_

To calculate the binding dissociation constant, K_D_ between the client protein and chaperone we used the formula

3 $${{{{{{\rm{K}}}}}}}_{{{{{{\rm{D}}}}}}}=\frac{{P}_{{{{{{\rm{chaperone}}}}}}}.{P}_{{{{{{\rm{client}}}}}}\; {{{{{\rm{protein}}}}}}}}{{P}_{{{{{{\rm{chaperone}}}}}}-{{{{{\rm{client}}}}}}\; {{{{{\rm{protein}}}}}}}}\cdot \frac{1}{V}\cdot \frac{1}{({6.022\times 10}^{23})\times {10}^{-27}}$$

where ${P}_{{{{{{\rm{chaperone}}}}}}-{{{{{\rm{client\; protein}}}}}}}$ is the probability that the chaperone and client protein are bound in the simulations, and ${P}_{{{{{{\rm{chaperone}}}}}}}$ and ${P}_{{{{{{\rm{client\; protein}}}}}}}$ are the probabilities, respectively, of unbound chaperone and unbound client protein configurations. V is the simulation volume. We converted this K_D_ to units of molarity, mol/L, by using the relevant conversion factor shown in Eq. 3. These probabilities were computed as the number of simulation frames the system was in a particular state divided by the total number of frames in the simulations. To assign frames to either bound or unbound states we used the following procedure: for each system, we plotted the time series of the total number of van der Waals contacts formed between the client protein and the chaperone. In most cases two-state behavior was observed, with a low number of inter-molecular contacts and then jumps to higher values, followed by a fall back to low numbers (Supplementary Fig. 17). We cross-referenced these events with a visualization of the simulation trajectory and found jumps to higher values corresponding to the client protein binding the apical domain of GroEL and inserting into the GroEL cavity. While the low values were transient interactions with the outside of GroEL. We then chose a threshold (Supplementary Table 9) separating these bound and unbound events and tested whether it was accurate by spot-checking whether other trajectories of the same system properly classified them as bound or unbound states. Thresholds for each system are shown as black horizontal lines in Supplementary Fig. 17.

### GroEL binding affinities for a spectrum of conformational states of a client protein

We generated different conformational states of client protein isochorismate synthase by performing high temperature (800 K) simulations followed by quenching to 310 K. We carried out 20 independent quenching simulations and then selected 20 different structures with distinct $Q$ and ${R}_{g}$ values across these different trajectories and then performed GroEL binding simulations with these 20 conformations (10 trajectories each) and calculated their K_D_ values. Each trajectory was simulated for 2.4 µs in the presence of GroEL and isochorismate synthase.

### All-atom simulations of GroEL and client proteins

We randomly chose one of the client proteins, isochorismate synthase, used in our coarse-grained simulations and simulated its interactions with GroEL at all-atom resolution. We chose ten representative structures from the coarse-grained ensembles of unfolded, folded, and near-native misfolded isochorismate synthase/GroEL systems and back-mapped these 30 coarse-grained structures to all-atom resolution using a previously reported procedure^28^. Next, each of these all-atom composite structures of the GroEL heptamer and client protein was solvated in a box of SPC/E water^97^ with dimensions 16 × 16 × 16 nm^3^ and then neutralized by the addition of 128 sodium ions. This neutralized system was then energy minimized with the steepest descent algorithm. Spherical harmonic restraints with a force constant 1000 kJ/(mol x nm^2^) were applied to the GroEL heptamer heavy atoms. All-atom simulations were carried out with GROMACS 2020^98^ using the AMBER03 force field^99^. Long-range electrostatic interactions were calculated with the Particle Mesh Ewald method^100^. Lennard-Jones interactions were calculated with a distance cut-off of 1.2 nm, and the temperature and pressure were maintained throughout the simulations at 310 K and 1 atm with a Nose-Hoover thermostat^101,102^ and Parrinello-Rahman barostat^103^, respectively. All bonds were constrained using the LINCS algorithm^104^ and an integration time step of 5 fs was used. We performed 1 ns of equilibration followed by a 1-ns production simulation with each of the 30 all-atom conformations before calculating the intermolecular interaction energies.

### Calculation of odds ratios of binding probabilities with and without attractive interactions between client proteins and chaperones

Odds ratios of the binding probabilities between chaperones and unfolded (U) or folded (F) conformations of client proteins with attractive van der Waals interactions on or off were calculated as

4 $${{{{{\rm{Odds}}}}}}\,{{{{{\rm{ratio}}}}}}=\frac{\left(\frac{{P}_{{{{{{\rm{U}}}}}},{{{{{\rm{on}}}}}}}}{{P}_{{{{{{\rm{U}}}}}},{{{{{\rm{off}}}}}}}}\right)}{\left(\frac{{P}_{{{{{{\rm{F}}}}}},{{{{{\rm{on}}}}}}}}{{P}_{{{{{{\rm{F}}}}}},{{{{{\rm{off}}}}}}}}\right)}$$

In Eq. 4, the terms ${P}_{{{{{{\rm{U}}}}}},{{{{{\rm{on}}}}}}}$ and ${P}_{{{{{{\rm{U}}}}}},{{{{{\rm{off}}}}}}}$ are the probabilities of protein/chaperone binding with the attractive interactions turned on or off, respectively, for unfolded client protein conformations. The terms ${P}_{{{{{{\rm{F}}}}}},{{{{{\rm{on}}}}}}}$ and ${P}_{{{{{{\rm{F}}}}}},{{{{{\rm{off}}}}}}}$ are the analogous values computed from simulations initialized with the client protein in the folded state. Odds ratios for interactions between misfolded or folded client protein conformations with chaperone interactions turned either on or off were computed using the equation

5 $${{{{{\rm{Odds}}}}}}\,{{{{{\rm{ratio}}}}}}=\frac{\left(\frac{{P}_{{{{{{\rm{M}}}}}},{{{{{\rm{on}}}}}}}}{{P}_{{{{{{\rm{M}}}}}},{{{{{\rm{off}}}}}}}}\right)}{\left(\frac{{P}_{{{{{{\rm{F}}}}}},{{{{{\rm{on}}}}}}}}{{P}_{{{{{{\rm{F}}}}}},{{{{{\rm{off}}}}}}}}\right)}$$

In Eq. 5, ${P}_{{{{{{\rm{M}}}}}},{{{{{\rm{on}}}}}}}$ and ${P}_{{{{{{\rm{M}}}}}},{{{{{\rm{off}}}}}}}$ are the binding probabilities of a misfolded client protein to chaperone with attractive van der Waal interactions turned on or off, respectively.

### Identification of entangled protein conformations

The six proteins whose interactions with GroEL/HtpG/DnaK we model here were previously identified to populate entangled conformations^27^ when they misfold. These entanglements are local non-covalent lasso entanglements that are not present in the native state that are associated with long-lived misfolded states within the *E. coli* proteome. We calculated the entanglement ($G$) of the native and near-native like misfolded states (Supplementary Table 17) based on a previously described method^27,34^. The code used to compute $G$ is available on GitHub at https://github.com/obrien-lab/topology_analysis. The value of $G$ is computed as

6 $$G=\frac{1}{N}\mathop{\sum}\limits_{\left(i,j\right)}\varTheta \left(\left(i,j\right)\in {{{{{\rm{nc}}}}}}\cap g\left(i,j\right)\ne {g}^{{{{{{\rm{native}}}}}}}\left(i,j\right)\right)$$

where $(i,j)$ is one of the native contacts in the native crystal structure; *nc* is the set of native contacts formed in the current structure; $g\left(i,j\right)$ and ${g}^{{{{{{\rm{native}}}}}}}\left(i,j\right)$ are, respectively, the total linking number of the native contact $(i,j)$ in the current and native structures; *N* is the total number of native contacts within the native structure; and the selection function $\varTheta$ equals 1 when the condition is true and 0 when it is false. The larger $G$ is the larger the number of residues that have changed their entanglement status relative to the native state. That is, $G$ reports on the presence of non-native entanglements in structures.

### Estimation of ATP consumption and state partitioning via a kinetic model

GroEL functions in a multi-step ATP-dependent cycle^25^. ATP first binds to GroEL before capturing an unfolded protein^105^. The GroES co-chaperone can then bind serving as a “lid.” Inside this GroES-enclosed GroEL cage, ATP hydrolysis and protein folding occur. The protein is released from the GroEL cage along with ADP and GroES. To simplify the GroEL-catalyzed protein refolding reaction we consider a single-ring reaction scheme shown in Supplementary Fig. 12. The folding of functionally active substrates by a single ring of GroEL/ES is possible^106,107^. In this reaction scheme, the GroEL heptamer binds 7 ATP molecules first, then binds to the unfolded protein, followed by GroES binding and ATP hydrolysis. After the 7 ATP molecules are hydrolyzed the protein is released in either the folded, misfolded, or still unfolded states, as are the GroES and ADP. Then free GroEL binds 7 ATP molecules again to start the next catalytic cycle. We assume that (1) the 7 ATP molecules bind simultaneously and can be treated as a single molecule and (2) only the unfolded protein can bind GroEL. The differential equations that describe this reaction scheme are the following:

7 $$\left\{\begin{array}{c}\frac{d[{{{{{\rm{G}}}}}}]}{dt}=-{k}_{1}\cdot [{{{{{\rm{G}}}}}}]\cdot [7{{{{{\rm{ATP}}}}}}]+{k}_{5}\cdot [{{{{{\rm{G}}}}}}|7{{{{{\rm{ADP}}}}}}|{{{{{\rm{U}}}}}}|{{{{{\rm{ES}}}}}}]+{k}_{8}\cdot [{{{{{\rm{G}}}}}}|7{{{{{\rm{ADP}}}}}}]\hfill \\ \frac{d[{{{{{\rm{G}}}}}}|7{{{{{\rm{ATP}}}}}}]}{dt}={k}_{1}\cdot [{{{{{\rm{G}}}}}}]\cdot [7{{{{{\rm{ATP}}}}}}]-{k}_{2}\cdot [{{{{{\rm{G}}}}}}|7{{{{{\rm{ATP}}}}}}]\cdot [{{{{{\rm{U}}}}}}]-{k}_{7}\cdot [{{{{{\rm{G}}}}}}|7{{{{{\rm{ATP}}}}}}]\hfill \\ \frac{d[{{{{{\rm{G}}}}}}|7{{{{{\rm{ATP}}}}}}|{{{{{\rm{U}}}}}}]}{dt}={k}_{2}\cdot [{{{{{\rm{G}}}}}}|7{{{{{\rm{ATP}}}}}}]\cdot [{{{{{\rm{U}}}}}}]-{k}_{3}\cdot [{{{{{\rm{G}}}}}}|7{{{{{\rm{ATP}}}}}}|{{{{{\rm{U}}}}}}]\cdot [{{{{{\rm{ES}}}}}}]\hfill \\ \frac{d[{{{{{\rm{G}}}}}}|7{{{{{\rm{ATP}}}}}}|{{{{{\rm{U}}}}}}|{{{{{\rm{ES}}}}}}]}{dt}={k}_{3}\cdot [{{{{{\rm{G}}}}}}|7{{{{{\rm{ATP}}}}}}|{{{{{\rm{U}}}}}}]\cdot [{{{{{\rm{ES}}}}}}]-{k}_{4}\cdot [{{{{{\rm{G}}}}}}|7{{{{{\rm{ATP}}}}}}|{{{{{\rm{U}}}}}}|{{{{{\rm{ES}}}}}}]\hfill \\ \frac{d[{{{{{\rm{G}}}}}}|7{{{{{\rm{ADP}}}}}}|{{{{{\rm{U}}}}}}|{{{{{\rm{ES}}}}}}]}{dt}={k}_{4}\cdot [{{{{{\rm{G}}}}}}|7{{{{{\rm{ATP}}}}}}|{{{{{\rm{U}}}}}}|{{{{{\rm{ES}}}}}}]-{k}_{5}\cdot [{{{{{\rm{G}}}}}}|7{{{{{\rm{ADP}}}}}}|{{{{{\rm{U}}}}}}|{{{{{\rm{ES}}}}}}]\hfill \\ \frac{d[{{{{{\rm{ES}}}}}}]}{dt}=-{k}_{3}\cdot [{{{{{\rm{G}}}}}}|7{{{{{\rm{ATP}}}}}}|{{{{{\rm{U}}}}}}]\cdot [{{{{{\rm{ES}}}}}}]+{k}_{5}\cdot [{{{{{\rm{G}}}}}}|7{{{{{\rm{ADP}}}}}}|{{{{{\rm{U}}}}}}|{{{{{\rm{ES}}}}}}]\hfill \\ \frac{d[7{{{{{\rm{ATP}}}}}}]}{dt}=-{k}_{1}\cdot [{{{{{\rm{G}}}}}}]\cdot [7{{{{{\rm{ATP}}}}}}]\hfill \\ \frac{d[{{{{{\rm{F}}}}}}]}{dt}={\varphi }_{F}^{GroEL}{k}_{5}\cdot [{{{{{\rm{G}}}}}}|7{{{{{\rm{ADP}}}}}}|{{{{{\rm{U}}}}}}|{{{{{\rm{ES}}}}}}]+{\varphi }_{F}^{Bulk}{k}_{6}\cdot [U]\hfill \\ \frac{d[{{{{{\rm{M}}}}}}]}{dt}={\varphi }_{M}^{GroEL}{k}_{5}\cdot [{{{{{\rm{G}}}}}}|7{{{{{\rm{ADP}}}}}}|{{{{{\rm{U}}}}}}|{{{{{\rm{ES}}}}}}]+{\varphi }_{M}^{Bulk}{k}_{6}\cdot [U]\hfill \\ \frac{d[{{{{{\rm{U}}}}}}]}{dt}=-{k}_{2}\cdot [{{{{{\rm{G}}}}}}|7{{{{{\rm{ATP}}}}}}]\cdot [{{{{{\rm{U}}}}}}]+(1-{\varphi }_{F}-{\varphi }_{M}){k}_{5}\cdot [{{{{{\rm{G}}}}}}|7{{{{{\rm{ADP}}}}}}|{{{{{\rm{U}}}}}}|{{{{{\rm{ES}}}}}}]-{k}_{6}\cdot [U]\hfill \\ \frac{d[{{{{{\rm{G}}}}}}|7{{{{{\rm{ADP}}}}}}]}{dt}={k}_{7}\cdot [{{{{{\rm{G}}}}}}]\cdot [7{{{{{\rm{ATP}}}}}}]-{k}_{8}\cdot [{{{{{\rm{G}}}}}}]\cdot [7{{{{{\rm{ADP}}}}}}]\hfill \end{array}\right.$$

where [G] is the concentration of single-ring GroEL; [G | 7ATP] is the concentration of the GroEL-ATP complex; [G | 7ADP] is the concentration of the GroEL-ADP complex; [G | 7ATP | U] is the concentration of the GroEL-ATP-unfolded protein complex; [G | 7ATP | U | ES] is the concentration of the GroEL-ATP-unfolded protein-GroES complex; [G | 7ADP | U | ES] is the concentration of the GroEL-ADP-unfolded protein-GroES complex; [ES] is the concentration of GroES; [7ATP] is the concentration of seven ATP molecules; [F] is the concentration of folded protein; [M] is the concentration of misfolded protein and [U] is the concentration of unfolded protein. The partition coefficients of the folded, misfolded and unfolded protein are ${\varphi }_{F}$, ${\varphi }_{M}$ and $1-{\varphi }_{F}-{\varphi }_{M}$ (see Supplementary Fig. 12). ${\varphi }_{F}^{{GroEL}}$ is the partition coefficient for GroEL assisted folding of the folded protein and ${\varphi }_{F}^{{Bulk}}$ is the partition coefficient for spontaneous folding in case of folded protein. ${\varphi }_{M}^{{GroEL}}$ is the partition coefficient for GroEL assisted folding for misfolded protein and ${\varphi }_{F}^{{Bulk}}$ is the partition coefficient for spontaneous folding in case of misfolded protein. The rate constants ${k}_{1}$ to ${k}_{8}$ are, respectively, the ATP binding rate, protein binding rate, GroES binding rate, ATP hydrolysis rate, protein release rate, spontaneous folding rate, basal hydrolysis rate and ADP release rate. These rate constants were taken from the literature^105,108–112^ and from Supplementary Data 1. For each experimental data set, we assign ${\varphi }_{F}$ from 0.001 to 1.001 with an interval of 0.001. And calculate ${\varphi }_{M}={P}_{{NN}}^{{eq}}/\left(1-{P}_{{NN}}^{{eq}}\right)\cdot {\varphi }_{F}$, where ${P}_{{NN}}^{{eq}}$ is the equilibrated probability of the non-native proteins obtained from the experimental data (the probability at the final time point). Note that we require ${\varphi }_{F}+{\varphi }_{M}\le 1$. Any ${\varphi }_{F}$ values that results in ${\varphi }_{F}+{\varphi }_{M} > 1$ is not used. We numerically solve the differential equations (Eq. 7) using the initial concentrations of GroEL, GroES, client protein and ATP reported in the original experiments (depicted in Supplementary Data 1). Then ${P}_{{NN}}^{{sim}}$ was calculated at the experimental time points as ${P}_{{NN}}^{{sim}}=1-\left[{{{{{\rm{F}}}}}}\right]/{\left[{{{{{\rm{U}}}}}}\right]}_{0}$, where ${\left[{{{{{\rm{U}}}}}}\right]}_{0}$ is the initial concentration of the client protein. The ${\varphi }_{F}$ and ${\varphi }_{M}$ values that maximize the Pearson correlation coefficient and minimize the absolute errors between ${P}_{{NN}}^{{sim}}$ and ${P}_{{NN}}^{\exp }$ are considered the best fit. The time course of [ATP] is then computed using these values.

### Generation of metastable structural states

We k-means clustered the last 100 ns of coarse-grained post-translational simulations resulting from 50 independent synthesis simulations^27,28^ along two order parameters that capture the nativeness of the structures (fraction of native contacts, $Q$) and the changes in self-entanglement of the protein (fraction of native contacts with a change in self-entanglement, G). These microstates are then coarse-grained into metastable states based on the PCCA + + algorithm^113^ . A set of representative structures for each metastable state were chosen by selecting at random from the five most probable microstates in each metastable state.

### Clustering of degenerate changes in self-entanglement

For each of these representative structures, we determine what native contacts have changes in their self-entanglement (relative to the native state) by examining changes in their partial linking number with the N or C terminus^27,28^. Furthermore, we determine the terminal tail residues located at the crossing of the loop plane formed by the native contacts (i.e. the residues that actually pierce the lasso loop) using Topoly^114^. If a loss of self-entanglement was observed (i.e. the loop is threaded in the native state but not in the misfolded state) we use the native state structure to find the crossing residues, else if it was a gain of self-entanglement we use the representative structure. We can then describe each individual change in self-entanglement in a structure by a discrete vector of 6 clustering parameters: (1) Number of crossings in the N-terminal change in entanglement; (2) Number of crossings in the C-terminal change in entanglement; (3) Rounded partial linking number for the N-terminal change in entanglement; (4) Rounded partial linking number for the C-terminal change in entanglement; (5) Change type for the N-terminal change in entanglement; (6) Change type for the C-terminal change in entanglement. The change type of a change in self-entanglement are described in detail in our previous work^27^. To ensure we are not clustering we also ensure that any entanglements clustered together by the above 6 parameters also have crossing residues within ±5 residues of each other. After clustering across all the changes in self-entanglement observed across all the representative metastable conformations, we then generate a list of representative changes in self-entanglement by choosing the entanglement with the minimal loop from each cluster (Supplementary Tables 12 and 13). We can then assign the representative changes in self-entanglement present in a given conformation to find the set of unique entangled states of the protein.

### Determining the statistical significance of the consistency between simulation data and experimental LiP-MS data

To answer the following questions: (1) how consistent are the changes in self-entanglement we observe with the experimentally observed changes in PK cut-site peptide abundance? (2) is this consistency more extreme than what you would expect with a random set of cut sites? We first quantify the consistency between our model and the experimental evidence by examining the existence of primary sequence overlap of the entangled residues with the PK cut-site residues and the consistency in the direction of change in solvent exposure of the PK cut-sites in the back-mapped representative MSM structures and LiP-MS data. We, therefore, define two test statistics for each time point $t$ as the average of two Boolean matrices for the overlap of the significant PK cut sites with changes in self entanglement ${\left\langle {{{{{\boldsymbol{O}}}}}}\right\rangle }_{t}$ and directional consistency in solvent exposure changes upon refolding of significant PK cut sites ${\left\langle {{{{{\boldsymbol{S}}}}}}\right\rangle }_{t}$. These matrices are of dimensions ${N}_{e}x{N}_{l}$, where ${N}_{e}$ is the number of unique representative entanglements in an entangled state and ${N}_{l}$ is the number of significant unique LiP-MS peptides across all time points.

8 $${O}_{e,l}=\left\{\begin{array}{cc}0 & {{\mbox{J}}}\left(l,e\right)=0\\ 1 & {{\mbox{J}}}\left(l,e\right) > 0\end{array}\right.{S}_{e,l}=\left\{\begin{array}{cc}0 & {{{{\mathrm{sgn}}}}}\left({\left\langle \Delta S\right\rangle }_{{sim}}\right)\ne {{{{\mathrm{sgn}}}}}\left({{{\log }}}_{2}R/N\right)\\ 1 & {{{{\mathrm{sgn}}}}}\left({\left\langle \Delta S\right\rangle }_{{sim}}\right)={{{{\mathrm{sgn}}}}}\left({{{\log }}}_{2}R/N\right)\end{array}\right.$$

Where ${{\mbox{J}}}\left(l,e\right)$ is the Jaccard index of a set of residues within ± 5 residues of a LiP-MS PK cut site, $l$, and the set of residues within 8 Å of the representative change in self-entanglements crossings, $e.$ If there is overlap (${O}_{e,l}=1$) we then use the sign function to determine if the direction of the average change in the solvent exposure of the PK cut-site residues we observe in given entangled state in our simulations, ${\left\langle \Delta S\right\rangle }_{{sim}}$, is the same as that of the experimentally observed peptide abundance ratio between refolded and native ensembles in the LiP-MS experiments for that residue.

If all the PK cut sites have overlap with representative changes in self-entanglement in a structure and the direction of change in the solvent exposure relative to the native state is the same that would indicate a complete consistency and both test statistics would be at their maximum. On the other hand, if none of the PK cut sites have overlap with representative changes in self-entanglement in a structure that would indicate a completely non-consistent result and both test statistics would be 0.

We employ the Permutation Test to determine the probability of observing the consistency between our model and the experimental data by random chance. For each experimental time point, we draw a random set of new PK cut-sites from a theoretical distribution of all potential half-tryptic peptides (those peptides cut by PK on one side and trypsin on the other). This is done in such a way that we maintain the same number of unique PK cut sites observed across all timepoints in the original experiment. We then calculate the new test statistics ${{\left\langle {{{{{\boldsymbol{O}}}}}}\right\rangle }_{t}}^{{\prime} }$ and ${{\left\langle {{{{{\boldsymbol{S}}}}}}\right\rangle }_{t}}^{{\prime} }$ and if there are two or more time points ${t}_{1}$ and ${t}_{2}$ where ${{\left\langle {{{{{\boldsymbol{O}}}}}}\right\rangle }_{t}}^{{\prime} } > {\left\langle {{{{{\boldsymbol{O}}}}}}\right\rangle }_{t}$ and ${{\left\langle {{{{{\boldsymbol{S}}}}}}\right\rangle }_{t}}^{{\prime} } > {\left\langle {{{{{\boldsymbol{S}}}}}}\right\rangle }_{t}$, and one of those time points is the longest experimental time point, we consider there to be more consistency between our model and this random set of significant PK cut sites than what we observed. We choose to add the additional criteria for the longest LiP-MS time point as we are interested in how consistent the long lived kinetically trapped misfolded state are. The p-value is then estimated as the probability of a randomly permutated set of significant PK cut sites having more consistency with the set of observed representative changes in self-entanglement than the experimentally observed set of significant LiP-MS peptides.

### Theoretical distribution of all potential PK cut-sites for random selection

As the LiP-MS data was analyzed across a proteome-wide analysis the data for each individual protein may suffer from a lack of coverage sufficient for random sampling. Therefore, we must generate a theoretical distribution of half-tryptic peptides. First calculating the intrinsic probability of PK cutting at a specific site across the proteome-wide set of data

9 $${P}_{{{{{{\rm{intrinsic}}}}}}}\left({AA}\right)=\frac{{P}_{{{{{{\rm{observed}}}}}}}\left({AA}\right)}{{P}_{{{{{{\rm{proteom}}}}}}}\left({AA}\right)}$$

Where ${P}_{{{{{{\rm{observed}}}}}}}\left({AA}\right)$ is the observed probability of a given AA being cut by PK and ${P}_{{{{{{\rm{proteom}}}}}}}\left({AA}\right)$ is the probability of AA across the proteome estimated from the protein databank. We then calculate the probability of observing a half-tryptic peptide with a given length and the number of internal trypsin cut sites across the proteome to control for the different time scales at which PK and trypsin are allowed to digest the protein (1 min for PK, 12 hrs for Trypsin). We then prepare a list of all possible half-tryptic peptides and randomly choose a peptide with replacement 10,000,000 times. For each iteration we generate two random number on the interval [0,1], one for the probability of a PK site being cut and the other for the probability of observing a peptide with a given length and a number of internal trypsin cut sites. If both of these random numbers are less than their respective probabilities than we accept the peptide into the theoretical set if it is not already present.

### Reporting summary

Further information on research design is available in the Nature Portfolio Reporting Summary linked to this article.

## Supplementary information

Supplementary Information

Description to Additional Supplementary Information

Dataset 1

Dataset 2

Supplementary Movie 1

Reporting Summary

## Data Availability

The data from mass spectrometry was previously published and deposited to the ProteomeXchange Consortium via the PRIDE partner repository with data set identifier PXD030869 [http://proteomecentral.proteomexchange.org/cgi/GetDataset?ID = PXD030869]. Summary data for these experiments are also provided in Supplementary Tables 10–13 and Supplementary Fig. 11. Structures used for the simulations in this study are: 1KP8 (GroEL), 1K7J (Transcription factor 1), 1A69 (purine nucleoside phosphorylase), 1P7L (S-adenosylmethionine synthetase), 2FYM (enolase), 3HWO (isochorismate synthase), 4A2C (galactitol-1-phosphate dehydrogenase), 5NRO (DnaK), 2IOQ (HtpG) and are freely available from the PDB. The processed data generated in this study are provided in the Source Data file and sample simulation trajectories of the systems studied here have been shown in Supplementary Movie 1. Source data are provided with this paper.

## Source data

Source Data

## Code availability

The OpenMM input files, Python (including the SciPy and PyEmma packages), Fortran and CHARMM scripts, and Visual Molecular Dynamics (VMD) v1.9.1 analysis codes, sample commands, ATP consumption codes, meta-analysis scripts, and molecular dynamics simulation scripts and example outputs and all related scripts are available in the GitHub repository under the accession code https://github.com/obrien-lab-psu/Subpopulations-of-soluble-misfolded-proteins-commonly-bypass-chaperones-How-it-happens-at-the-mole. All the software used for the experimental data analysis in this study is available in the GitHub repository under the accession code https://github.com/FriedLabJHU/Refoldability-Tools.
