# Supplementary material for: How soluble misfolded proteins bypass chaperones at the molecular level
Source: Nat Commun. 2023 Jun 21;14:3689. doi: 10.1038/s41467-023-38962-z (PMC10284856; doi:10.1038/s41467-023-38962-z)
Supplement: Supplementary file 1 — Supplementary Information [file 41467_2023_38962_MOESM1_ESM.pdf]

Supporting Information

How soluble misfolded proteins bypass chaperones at the molecular level

Ritaban Halder[1, a], Daniel A. Nissley[1, a, b], Ian Sitarik[1, a], Yang Jiang[1], Yiyun Rao[2], Quyen. V. Vu[3], Mai Suan Li[3,4], Justin Pritchard[5,6] and Edward P. O'Brien[1, 7, 8*]

[1] Department of Chemistry, Pennsylvania State University, University Park, PA 16802, USA

[2] Molecular, Cellular and Integrative Biosciences Program, The Huck Institutes of the Life Sciences, Pennsylvania State University, University Park, PA 16802, USA

[3]Institute of Physics, Polish Academy of Sciences; Al. Lotnikow 32/46, 02-668 Warsaw, 6 Poland

[4]Institute for Computational Sciences and Technology; Quang Trung Software City, Tan 13 Chanh Hiep Ward, District 12, Ho Chi Minh City, Vietnam

[5]Department of Biomedical Engineering, Pennsylvania State University, State College, PA 16802, USA

[6] Huck Institute for the Life Sciences, Pennsylvania State University, State College, PA 16802, USA.

[7]Bioinformatics and Genomics Graduate Program, The Huck Institutes of the Life Sciences, Pennsylvania State University, University Park, PA 16802, USA

[8]Institute for Computational and Data Sciences, Pennsylvania State University, University Park, PA 16802, USA

[a]These authors contributed equally

[b]Current Address: Department of Statistics, University of Oxford, Oxford, OX1 3LB, UK.

* to whom correspondence should be addressed: epo2@psu.edu

Supplementary Note 1. Reaction scheme for kinetic partitioning model.

Consider a three-state folding kinetics with parallel pathways as shown below:

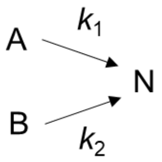

It describes the condition where state A and B can both fold to the native state N with a rate of k_1 and k_2, respectively, and there are no transitions between A and B. Let's assume that $k_1 > k_2$, which means state A is a fast-folding species and state B is a slow-folding species. State A and B have occupied a_0 and a_1 of the initial population of the post-translational folding, respectively, and a_0+$a_1 = 1$. Therefore, the time evolution of the population [A] and [B] will be

$$\begin{cases} \frac{\mathrm{d[A]}}{\mathrm{d}t} = -k_1[\mathrm{A}] \\ \frac{\mathrm{d[B]}}{\mathrm{d}t} = -k_2[\mathrm{B}] \end{cases} \quad \text{(Eq. 10)}$$

Solving the ordinary differential equations with the initial condition $[\mathrm{A}]_0 = a_0$ and $[\mathrm{B}]_0 = a_1$, we obtain the survival probability of the non-native species:

$$S_U(t) = [\mathrm{A}] + [\mathrm{B}] = a_0 \exp(-k_1 t) + a_1 \exp(-k_2 t) \quad \text{(Eq. 11)}$$

Supplementary Table 1. Results of fitting Eq. 1 to the 20 experimental refolding time courses

Label of plots in Supplementary Figure 1	Ref.	a_0	k_1 (min^{-1})	a_1	k_2 (min^{-1})	Pearson R^2
a	5	0.43	0.91	0.57	2×10^{-3}	0.98
b	6	0.81	0.37	0.19	1×10^{-4}	0.98
c	7	0.92	0.06	0.08	9×10^{-22}	0.91
d	8	0.57	0.02	0.43	1×10^{-16}	0.98
e	9	0.74	0.06	0.26	2×10^{-16}	0.99
f	10	0.50	0.01	0.50	4×10^{-18}	1.00
g	11	0.65	0.15	0.35	1×10^{-2}	1.00
h	12	0.74	0.06	0.26	1×10^{-19}	0.99
i	13	0.73	0.18	0.27	5×10^{-23}	0.98
j	14	0.83	0.10	0.17	9×10^{-3}	1.00
k	15	0.62	24.38†	0.38	8×10^{-2}†	0.95
l	16	0.30	0.11	0.70	4×10^{-20}	0.99
m	17	0.60	0.07	0.40	9×10^{-19}	1.00
n	18	0.90	0.14	0.10	1×10^{-12}	0.99
o	19	0.77	0.16	0.23	2×10^{-23}	0.97
p	20	0.75	0.14	0.25	3×10^{-3}	0.99
q	21	0.57	0.01	0.43	2×10^{-22}	0.99
r	22	0.86	0.16	0.14	3×10^{-14}	0.98
s	23	0.95	0.02	0.05	1×10^{-13}	1.00
t	24	0.80	0.09	0.20	3×10^{-20}	1.00

†These are the only rates in this table that use units of hour^{-1} as compared to min^{-1}.

Supplementary Table 2. ATP consumption analysis using mathematical modelling of GroEL-GroES assisted protein folding process in our meta-analysis.

**Initial ATP concentration (**μM)	**ATP consumption during experiment (**μM)	**Reference No**
5000	340	5
2000	42	6
10000	380	14
2000	107	18
1000	50	19
5000	600	20
5000	50	22
4000	2000	23
1000	350	24

Supplementary Table 3. $\varphi_F(\text{Bulk})$ and $\varphi_M(\text{Bulk})$ estimated from the available spontaneous refolding data in the absence of GroEL (Eq. 7 and Supplementary Figure 12) are compared with φ_F and φ_M. Dashed lines mean no data was available.

Reference No	Protein name	$\boldsymbol{\varphi_F}$	$\boldsymbol{\varphi_M}$	$\boldsymbol{\varphi_U}$	$\boldsymbol{\varphi_F}$ **(Bulk)**	$\boldsymbol{\varphi_M}$ **(Bulk)**
5	Aconitase	0.032	0.036	0.932	0.14	0.86
6	PepQ	0.065	0.014	0.921	0.56	0.44
14	Rhodanese	0.014	0.001	0.985	-	-
18	Rubisco	0.026	0.002	0.972	-	-
19	Rubisco	0.031	0.008	0.961	0.03	0.97
20	Interferon Gamma	0.014	0.003	0.983	-	-
22	Rubisco	0.035	0.006	0.959	0.04	0.96
23	Malate dehydrogenase	0.004	0.002	0.994	0.04	0.96
24	Rhodanese	0.012	0.003	0.985	0.03	0.97

Supplementary Table 4. The percentage of trajectories that misfold and involve entanglements for (taken from Ref. 27).

Client protein	PDB ID	% of misfolded trajectories	% of misfolded trajectories that involve lasso topologies
Isochorismate synthase	3HWO	48%	100%
Enolase	2FYM	34%	100%
Galactitol-1-phosphate-dehydrogenase	4A2C	20%	100%
Protein Transcription factor 1	1K7J	68%	26%
S-adenosylmethionine synthetase	1P7L	94%	100%
Purine nucleoside phosphorylase	1A69	36%	100%

Supplementary Table 5. Average K_D of different client protein to chaperone in unfolded, misfolded and folded states (Eq. 6). Upper and lower bounds of the 95% confidence interval (CI) are listed in parentheses.

Client protein	Chaperone	Conformational State	K_D (95% CI), μM
Isochorismate synthase	GroEL	Unfolded<br>Misfolded<br>Folded	86.6 (60.6:113)<br>553 (386:720)<br>500 (108:892)
Enolase	GroEL	Unfolded<br>Misfolded<br>Folded	39.1 (13.7:64.5)<br>726 (611:841)<br>557 (464:650)
Galactitol-1-phosphate-dehydrogenase	GroEL	Unfolded<br>Misfolded<br>Folded	88.6 (59.4:118)<br>391(212:570)<br>790 (577:1000)
Protein Transcription factor 1	GroEL	Unfolded<br>Misfolded<br>Folded	34.7 (20.1:49.3)<br>414 (252:576)<br>698 (404:992)
S-adenosylmethionine synthetase	GroEL	Unfolded<br>Misfolded<br>Folded	84.9 (57.1:113)<br>443 (322:564)<br>618 (363:873)
Purine nucleoside phosphorylase	GroEL	Unfolded<br>Misfolded<br>Folded	112 (83.4:141)<br>534 (135:933)<br>472 (422:523)
Purine nucleoside phosphorylase	HtpG	Unfolded<br>Misfolded<br>Folded	24.8 (16.8:32.8)<br>219 (168:270)<br>234 (196:272)
Purine nucleoside phosphorylase	DnaK	Unfolded<br>Misfolded<br>Folded	25.2 (17.9:32.5)<br>364 (230:498)<br>286 (214:359)

Supplementary Table 6. p-values for the difference in K_D between unfolded ($K_{D,U}$) and folded ($K_{D,F}$) states, and misfolded ($K_{D,M}$) versus folded ($K_{D,F}$) states calculated using a two-tailed permutation test (n = 1,000,000).

**Client protein**	**Chaperone**	**p-value ($K_{D,U}$ vs $K_{D,F}$)**	**p-value ($K_{D,M}$ vs $K_{D,F}$)**
Isochorismate synthase	GroEL	8.4×10^{-5}	0.88
Enolase	GroEL	1.1×10^{-5}	0.038
Galactitol-1-phosphate-dehydrogenase	GroEL	1.2×10^{-5}	0.012
Protein Transcription factor 1	GroEL	9.9×10^{-7}	0.11
S-adenosylmethionine synthetase	GroEL	1.5×10^{-5}	0.25
Purine nucleoside phosphorylase	GroEL	3.0×10^{-6}	0.92
Purine nucleoside phosphorylase	HtpG	1.2×10^{-5}	0.63
Purine nucleoside phosphorylase	DnaK	9.9×10^{-6}	0.23

Supplementary Table 7. Binding probabilities of unfolded, misfolded, and folded states of client proteins for GroEL in the presence and absence of attractive interactions. Note that, the effective odd's ratio as shown in Table 3, is actually the ratio of binding probability between client protein and GroEL in the presence of attractive interactions at different conformation since the binding probabilities between client protein and GroEL in the absence of attractive interactions are both zero and cancel out. The binding probability of each of the unfolded, misfolded and folded states for each client protein and GroEL was averaged over ten independent simulations.

		**In presence of attractive interaction**			**In absence of attractive interaction**
**Client protein**	**PDB ID**	**Binding probability (Unfolded state)**	**Binding probability (Misfolded state)**	**Binding probability (Folded state)**	**Binding probability (Unfolded, Misfolded and Folded State**
Isochorismate synthase	3HWO	0.42	0.15	0.20	0.00
Purine nucleoside phosphorylase	1A69	0.38	0.19	0.15	0.00
Galactitiol-1-phosphate dehydrogenase	4A2C	0.43	0.19	0.11	0.00
S-adenosyl methionine synthetase	1P7L	0.43	0.17	0.15	0.00
Enolase	2FYM	0.58	0.11	0.13	0.00
Transcription factor 1	1K7J	0.59	0.18	0.13	0.00

Supplementary Table 8. Percentage of trapped trajectories starting from entangled (misfolded) and not-entangled (folded) structures of Isochorismate Synthase taken from Supplementary Figure 3.

Client protein	Entanglement status of starting structure	Structure index	% of trapped trajectories
Isochorismate Synthase	Not entangled	1	10
		2	0
		3	0
		4	0
		5	0
	Entangled	1	100
		2	100
		3	100
		4	100

Supplementary Table 9. Contact thresholds used to classify client proteins as either bound or unbound to a chaperone. See also Supplementary Figure 4.

Client protein	Chaperone	Contact threshold
Isochorismate synthase	GroEL	150
Enolase	GroEL	150
Galactitol-1-phosphate-dehydrogenase	GroEL	175
Protein Transcription factor 1	GroEL	75
S-adenosylmethionine synthetase	GroEL	140
Purine nucleoside phosphorylase	GroEL	120
Purine nucleoside phosphorylase	HtpG	20
Purine nucleoside phosphorylase	DnaK	20

Supplementary Table 10. Summary of LiP-MS peptides with a statistically significant change in their abundance upon refolding.

**S-adenosylmethionine synthetase (**P0A817) **Cytosolic Buffer**			**Enolase (**P0A6P9) **Cytosolic Buffer**		
**1min**	**5min**	**2hr**	**1min**	**5min**	**2hr**
---	A2	A2	P129*	P129*	P129*
---	S60*	S60*	M151*	M151*	M151*
A61	---	---	I153	---	---
W62	---	---	M170*	---	M170*
V63	---	---	G186	---	---
---	F124	---	---	---	L193
T128	---	---	N203	---	---
---	---	N129	A227	A227	---
---	---	A214	---	G234	---
---	S310	---	---	S270	S270
G317*	G317*	G317*	A359	---	---
Y339	Y339	---	G363*	G363*	G363*
---	Q343*	Q343*	---	D382	---
M344	---	---	---	L383*	L383*
			---	---	I410
			E414	---	---

*PK cut sites consistent with our misfolded conformations and present at the longest refolding time point and at least one other time point.

Supplementary Table 11. Representative changes in self-entanglement for S-adenosyl methionine synthetase.

index	NC	Termini	Ref partial linking	Frame partial linking	Crossing residue	Residues surrounding crossings
0	23, 353	N	0.489	0.616	10	8, 9, 10, 11, 12, 139, 142, 143, 146, 166, 167, 168, 249
1	36, 348	C	0.132	-0.626	365	125, 126, 127, 128, 129, 363, 364, 365, 366, 367, 368, 369, 370
2	43, 265	C	0.16	-0.663	299	115, 116, 117, 118, 119, 120, 121, 122, 123, 124, 275, 297, 298, 299, 300, 301
3	45, 243	C	0.423	-0.623	251	120, 121, 122, 248, 249, 250, 251, 252, 253, 359, 360, 361
4	48, 237	C	0.397	-0.662	300, 263	117, 118, 119, 120, 121, 298, 299, 300, 301, 302, 310, 311, 336, 247, 261, 262, 263, 264, 265, 266, 272, 273, 356
5	48, 240	N	0.639	0.252	42, 21	24, 25, 27, 28, 40, 41, 42, 43, 44, 53, 54, 55, 56, 57, 96, 264, 265, 266, 17, 18, 19, 20, 21, 22, 23, 24, 25, 44, 242, 243, 244, 264, 265, 356, 357
6	78, 163	N|C	0.613 | 0.718	0.225 | -0.015	40, 21, 56, 264	28, 38, 39, 40, 41, 42, 56, 57, 58, 59, 265, 266, 267, 17, 18, 19, 20, 21, 22, 23, 24, 25, 44, 242, 243, 244, 264, 265, 356, 357, 40, 41, 42, 54, 55, 56, 57, 58, 96, 97, 98, 99, 21, 22, 24, 25, 42, 243, 244, 262, 263, 264, 265, 266, 273, 352, 356
7	83, 162	C	0.72	-0.02	264	21, 22, 24, 25, 42, 243, 244, 262, 263, 264, 265, 266, 273, 352, 356
8	134, 284	N	1.042	0.033	10	8, 9, 10, 11, 12, 139, 142, 143, 146, 166, 167, 168, 249, 253, 358
9	257, 296	N	0.724	-0.097	124	122, 123, 124, 125, 126, 256, 257, 258, 259, 276, 279, 280, 296, 297, 298, 299
10	300, 310	N	0.012	-0.855	115	113, 114, 115, 116, 117, 300, 301, 302, 303, 304, 305, 306, 307, 309, 337
11	23, 353	N	0.489	0.616	10	8, 9, 10, 11, 12, 139, 142, 143, 146, 166, 167, 168, 249,

Supplementary Table 12. Representative changes in self-entanglement for Enolase.

index	NC	Tail	Ref partial linking	Frame partial linking	Crossing residue	Residues surrounding crossings
0	16, 208	C	0.802	-0.318	386, 373, 396	, 130, 131, 132, 133, 134, 135, 143, 144, 145, 382, 383, 384, 385, 386, 387, 388, 389, 14, 15, 16, 18, 38, 39, 40, 208, 371, 372, 373, 374, 375, 151, 152, 153, 186, 190, 209, 210, 211, 394, 395, 396, 397, 398, 402, 403
1	23, 114	N	0.159	-0.632	9	7, 8, 9, 10, 11, 21, 22, 23, 24, 68, 69, 71, 72,
2	40, 371	C	0.482	-0.606	386	, 130, 131, 132, 133, 134, 135, 142, 143, 144, 145, 381, 382, 383, 384, 385, 386, 387, 388
3	138, 353	C	1.232	0.127	367	, 338, 339, 340, 341, 365, 366, 367, 368, 369, 389, 390, 391, 392, 393
4	147, 384	C	0.755	-0.054	391	, 146, 147, 148, 149, 150, 366, 367, 368, 384, 389, 390, 391, 392, 393, 427, 428
5	155, 168	C	-0.008	-0.968	397	, 151, 152, 153, 154, 155, 156, 157, 158, 159, 162, 163, 164, 165, 166, 190, 191, 395, 396, 397, 398, 399
6	177, 423	N	0.641	-0.225	168, 154	152, 153, 154, 155, 156, 157, 159, 166, 167, 168, 169, 170, 245, 246, 247, 152, 153, 154, 155, 156, 168, 169, 170, 211, 212, 213, 214, 222, 226
7	183, 406	N | C	-0.07 | 0.033	-0.804 | -0.635	170, 419	150, 151, 152, 153, 154, 168, 169, 170, 171, 172, 243, 244, 245, 246, 143, 144, 392, 393, 396, 397, 413, 416, 417, 418, 419, 420, 421
8	190, 396	N	0.016	-0.856	169, 141, 151	152, 153, 154, 155, 166, 167, 168, 169, 170, 171, 222, 223, 243, 244, 245, 246, 132, 135, 136, 139, 140, 141, 142, 143, 364, 365, 366, 367, 368, 386, 149, 150, 151, 152, 153, 170, 171, 172, 186, 393, 394, 395, 396, 397
9	322, 339	C	-0.05	-0.907	358	, 333, 334, 335, 336, 337, 338, 354, 355, 356, 357, 358, 359, 360
10	356, 366	N	0.058	-0.934	338	336, 337, 338, 339, 340, 356, 357, 358, 359, 360, 361, 362, 363, 364, 365, 366
11	381, 405	N	0.222	-0.795	147	145, 146, 147, 148, 149, 384, 385, 388, 389, 390, 391, 392, 399, 400, 401, 402, 403
12	381, 409	N | C	0.283 | 0.057	-0.777 | -0.615	144, 420	142, 143, 144, 145, 146, 385, 386, 387, 388, 419, 420, 143, 144, 145, 146, 385, 412, 413, 414, 416, 417, 418, 419, 420, 421, 422
13	382, 409	N | C	0.303 | 0.084	-0.79 | 0.616	144	142, 143, 144, 145, 146, 385, 386, 387, 388, 389, 419, 420, 421
14	390, 423	N	1.092	0.031	146	144, 145, 146, 147, 148, 178, 179, 384, 385, 388, 390, 391, 409, 413, 420, 421, 422, 423
15	394, 405	N	0.128	-0.862	163	161, 162, 163, 164, 165, 395, 396, 397, 398, 399, 400, 401, 402, 403
16	394, 405	N	0.128	-0.935	149	147, 148, 149, 150, 151, 172, 173, 391, 392, 393, 394, 397, 398, 399, 400, 401, 402, 403, 404

Supplementary Table 13. Summary of one-sided permutation test (n=100,000) results for significant consistency between simulation and experimental data. Null distribution for hypothesis testing was constructed as described in the methods. All p-values are corrected using the FDR multiple hypothesis correction method.

Ent State ID	S-adenosyl methionine synthetase				Enolase			
	p-value	<Q>	<G>	Rep ent ID	p-value	<Q>	<G>	Rep ent ID
0	1.000	0.759	0.099	[5, 6, 7, 8]	0.058	0.921	0.065	[0, 3, 4, 6, 7, 11, 12, 13, 14, 15]
1	0.169	0.728	0.067	[5, 7, 8, 9]	0.069	0.871	0.066	[3, 4, 6, 7, 11, 12, 14, 15]
2	1.000	0.759	0.059	[7]	**0.002**	**0.902**	**0.063**	**[2, 3, 4, 5, 11, 15]**
3	1.000	0.762	0.070	[1, 7]	**0.040**	**0.916**	**0.061**	**[0, 3, 6, 8, 11, 14, 15]**
4	0.169	0.780	0.067	[1, 7, 9]	**0.033**	**0.897**	**0.062**	**[3, 6, 11, 14, 15]**
5	**0.018**	**0.839**	**0.163**	**[2, 4, 7, 8, 10]**	0.120	0.920	0.028	[0, 3, 9]
6	**0.018**	**0.831**	**0.145**	**[2, 7, 8, 10]**	0.058	0.878	0.025	[3, 10]
7	0.766	0.791	0.165	[0, 2, 4, 7, 8, 10]	**0.043**	**0.888**	**0.028**	**[3]**
8	0.169	0.870	0.097	[7, 8, 9, 10]	0.207	0.924	0.025	[0, 3, 10]
9	1.000	0.877	0.094	[7, 8, 10]	**0.022**	**0.921**	**0.084**	**[3, 4, 5, 6, 14, 15, 16]**
10	0.039	0.844	0.094	[3, 7, 8, 10]	**0.041**	**0.916**	**0.080**	**[0, 2, 3, 4, 5, 6, 14, 15, 16]**
11					**0.023**	**0.921**	**0.067**	**[0, 2, 3, 4, 5, 14, 15, 16]**
12					**0.002**	**0.936**	**0.082**	**[0, 3, 4, 5, 6, 14, 15, 16]**
13					1.000	0.908	0.036	[0, 3, 9, 10]
14					1.000	0.914	0.037	[3, 9, 10]
15					0.120	0.925	0.113	[3, 4, 5, 6, 8, 14, 15]
16					**0.009**	**0.921**	**0.102**	**[0, 2, 3, 4, 5, 6, 8, 14, 15]**
17					**0.000**	**0.914**	**0.104**	**[0, 3, 4, 5, 6, 8, 14, 15]**
18					1.000	0.927	0.001	[1]

*****Significant *p*-values < 0.05 are displayed in bold

Supplementary Table14. Identities of the client proteins and their corresponding η values used to tune the native state stability (see Methods).

Client protein	PDB ID	Domains and interfaces	Optimum η used for coarse-grained modelling of client proteins
Isochorismate synthase	3HWO	Domain 1: 1-391	1.359
Enolase	2FYM	Domain 1: 1-127	1.359
		Domain 2: 128-432	1.114
		1|2 Interface	2.480
Galactitol-1-phosphate-dehydrogenase	4A2C	Domain 1: 1-346	1.359
Transcription factor 1	1K7J	Domain 1: 1-206	1.114
S-Adenosylmethionine synthetase	1P7L	Domain 1: 1-10; 137-233	1.114
		Domain 2: 11-105; 234-270	1.359
		Domain 3: 106-136; 271-384	1.114
		1|2 Interface	1.507
		1|3 Interface	1.235
		2|3 Interface	2.480
Purine nucleoside phosphorylase	1A69	Domain 1: 1-239	1.359

Supplementary Table 15. Identities of the chaperones and their corresponding η values (see Methods).

Chaperone	PDB ID	η used for coarse-grained modelling
GroEL	1KP8	1.8
HtpG	2IOQ	1.4
DnaK	5NRO	1.4

Supplementary Table 16. η values for scaling Lennard-Jones well-depths between client proteins and chaperones (see Methods)

**Chaperone-Client protein Complex**	**η used for chaperone-client protein binding simulations**
GroEL-Isochorismate synthase	0.160
GroEL-Enolase	0.155
GroEL-Galactitol-1-phosphate Dehydrogenase	0.153
Groel-Transcription factor 1	0.140
GroEL-S-Adenosine methionine Synthetase	0.160
Groel-Purine nucleoside phosphorylase	0.150
HtpG-Purine nucleoside phosphorylase	0.150
DnaK-Purine nucleoside phosphorylase	0.160

Supplementary Table 17. Entanglement (G, Eq. 6) calculated for the native and near-native like misfolded states of client proteins.

Client protein Name	Entanglement in the native state?	G	Entanglement present in near-native misfolded state?	G
Isochorismate synthase	No	0.00	Yes	0.05
Enolase	No	0.00	Yes	0.09
Galactitol-1-phosphate-dehydrogenase	No	0.00	Yes	0.01
Transcription factor 1	No	0.00	Yes	0.10
S-adenosylmethionine synthetase	No	0.00	Yes	0.08
Purine nucleoside phosphorylase	No	0.00	Yes	0.09

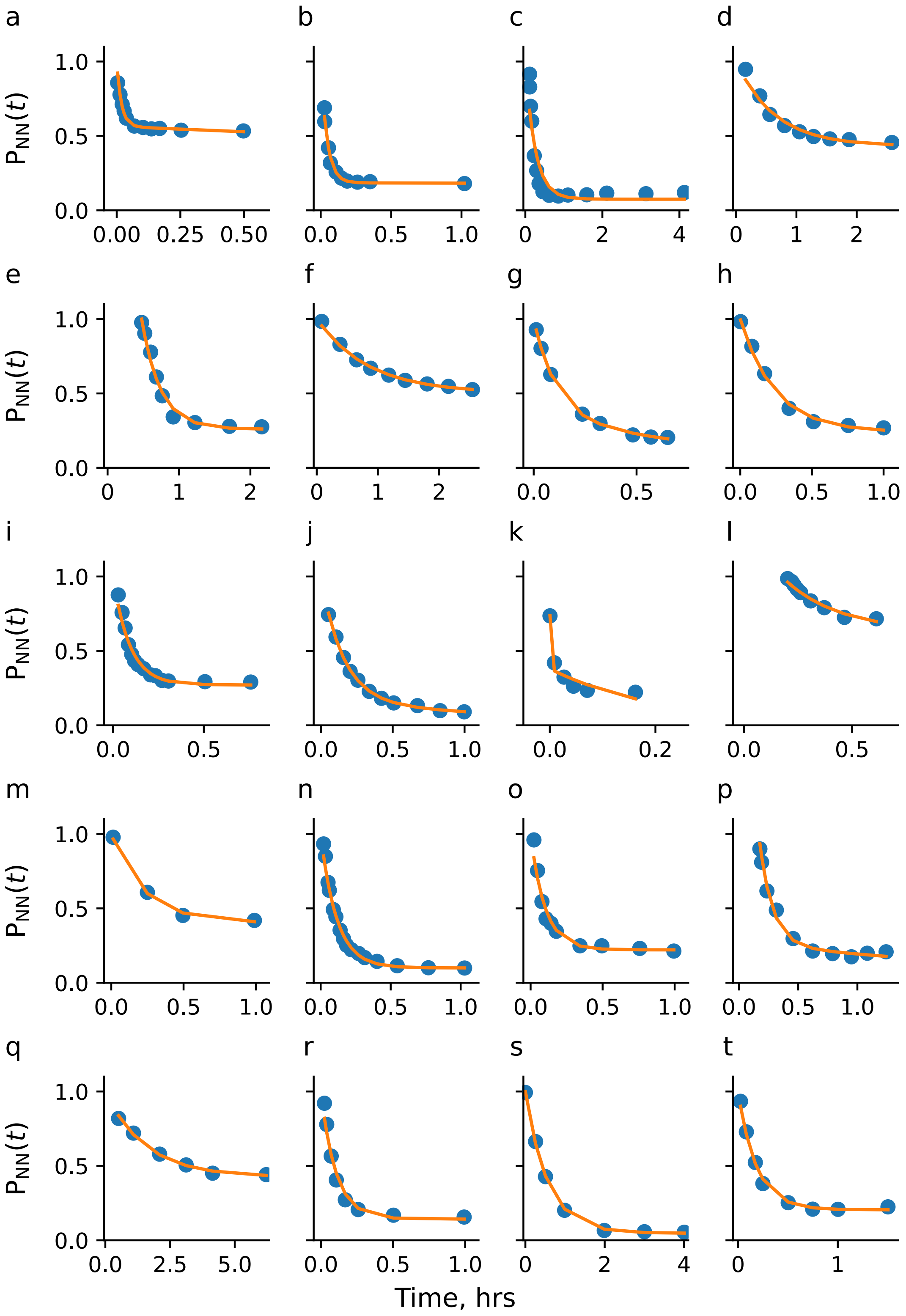

Supplementary Figure 1. Curve-fitting results from the 20 experimental refolding studies. Values of $P_{\mathrm{NN}}(t)$ (orange line) computed as described in the Methods from the original experimental data (blue points) were fit to Eq. 1 using SciPy in Python 3.

The resulting fit parameters and Pearson R^2 values are listed in Supplementary Table 1 while the original figures from which data were extracted with PlotDigitizer are reproduced in Supplementary Data 1.

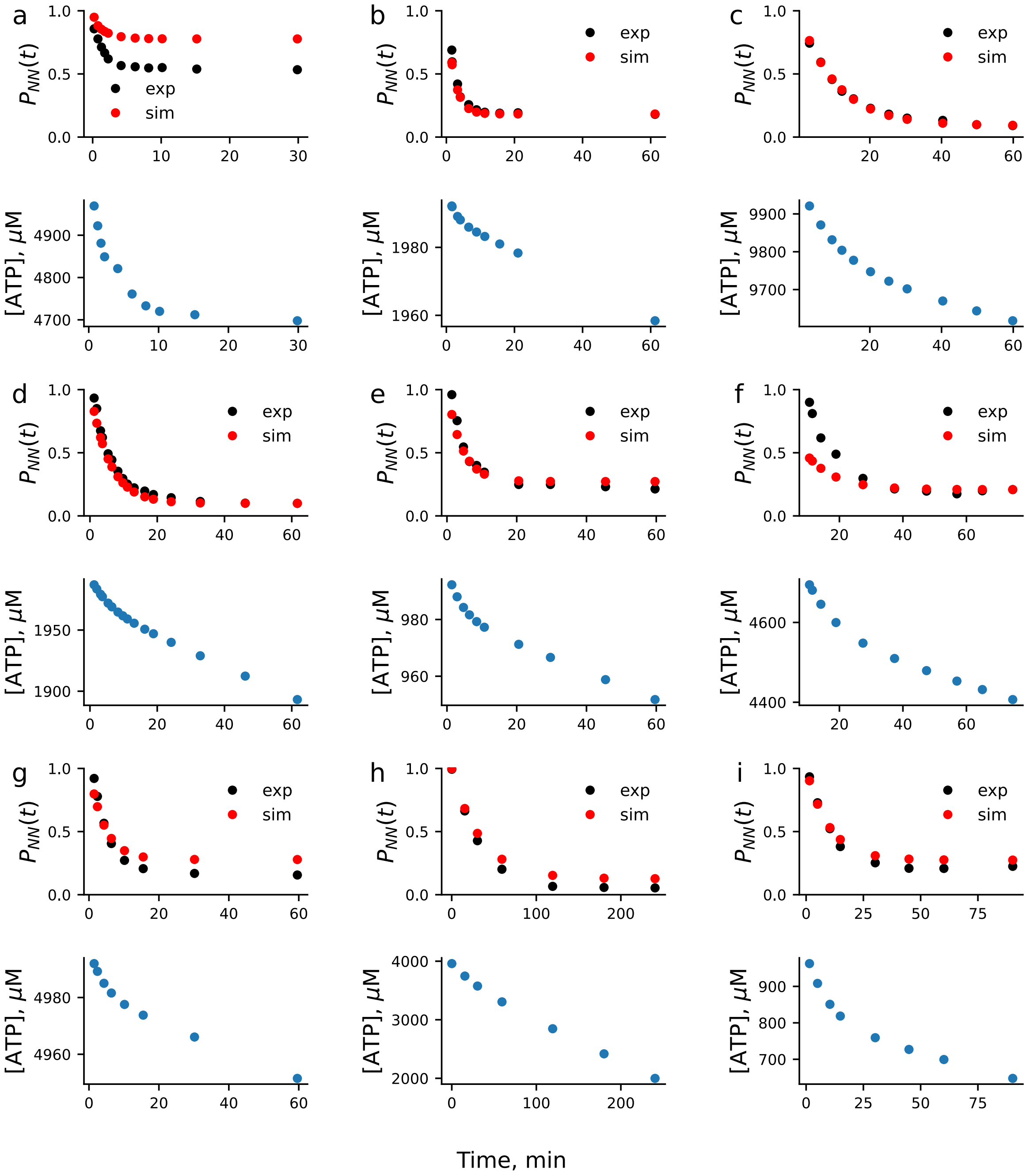

Supplementary Figure 2. Experimental and simulated probability of non-native state formation and corresponding ATP consumption analysis using our model (Eq. 7). The probability of non-native state formation (experimental and simulated) as well as the ATP consumption of the GroEL-GroES assisted meta-analysis in our study for (a) Aconitase, (b) PepQ, (c, i) Rhodanese, (d, e, g) Rubisco, (f) Interferon Gamma, (h) Malate dehydrogenase. The list of those references which utilizes GroEL-GroES chaperone in our studied meta-analysis were depicted in the Supplementary Data 1.

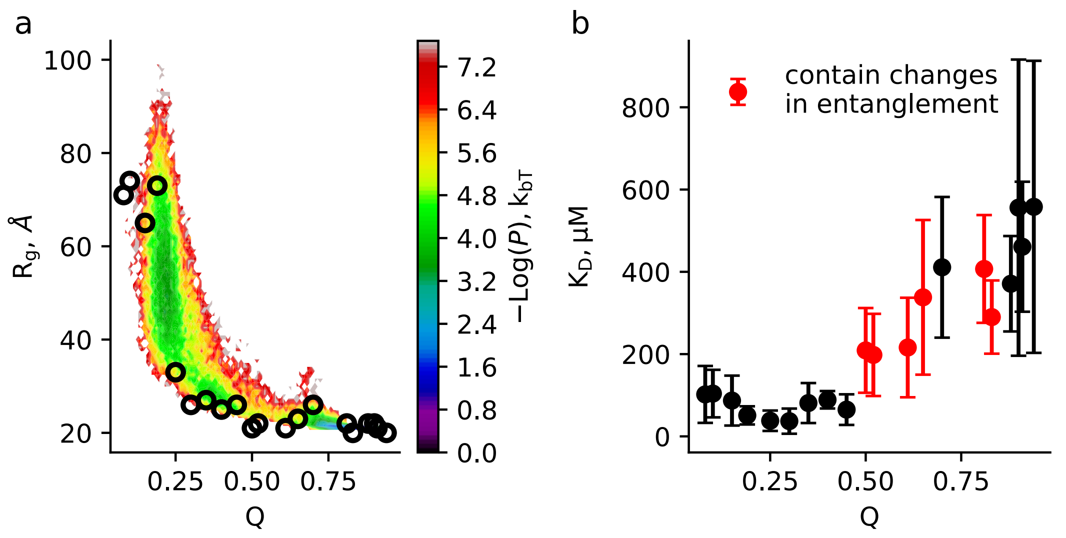

Supplementary Figure 3. Sampling different non-native states and their corresponding binding dissociation constants with GroEL. (a) Q vs R_g Log-probability plot of isochorismate synthase starting from an unfolded to folded state. Black circles indicate the values of 20 selected client protein structures. Some have low populations and not readily apparent on the plot due to visualization approximations. (b) K_D versus Q for the 20 selected client protein conformations (from (a)) with GroEL. The red circles in the plot indicate states containing a misfolded entanglement, other points do not. Error bars are 95% confidence intervals about the mean values of 10 independent simulations of each protein conformation.

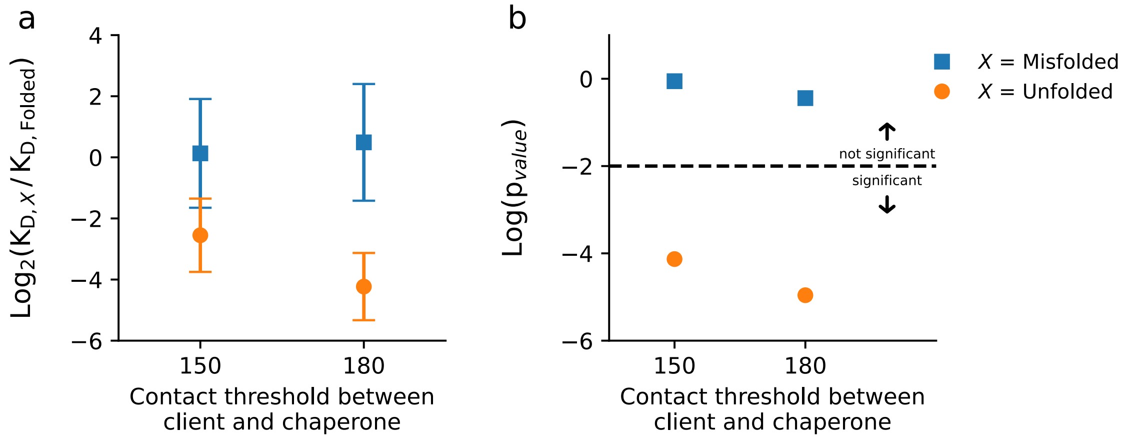

Supplementary Figure 4. Influence of different contact thresholds on GroEL/Isochorismate synthase binding. (a) Dissociation constant (logK_D) when a contact threshold of ≥150 residues and ≥180 is used. Error bars are 95% confidence intervals about the mean values of 10 independent simulations. (b) Log(p_{value}) indicates ($K_{D,Misfolded}/K_{D,Folded}$) is not significant whereas ($K_{D,Unfolded}/K_{D,Folded}$) is significant. p-values were computed using a two-tailed Permutation Test (n=1,000,000).

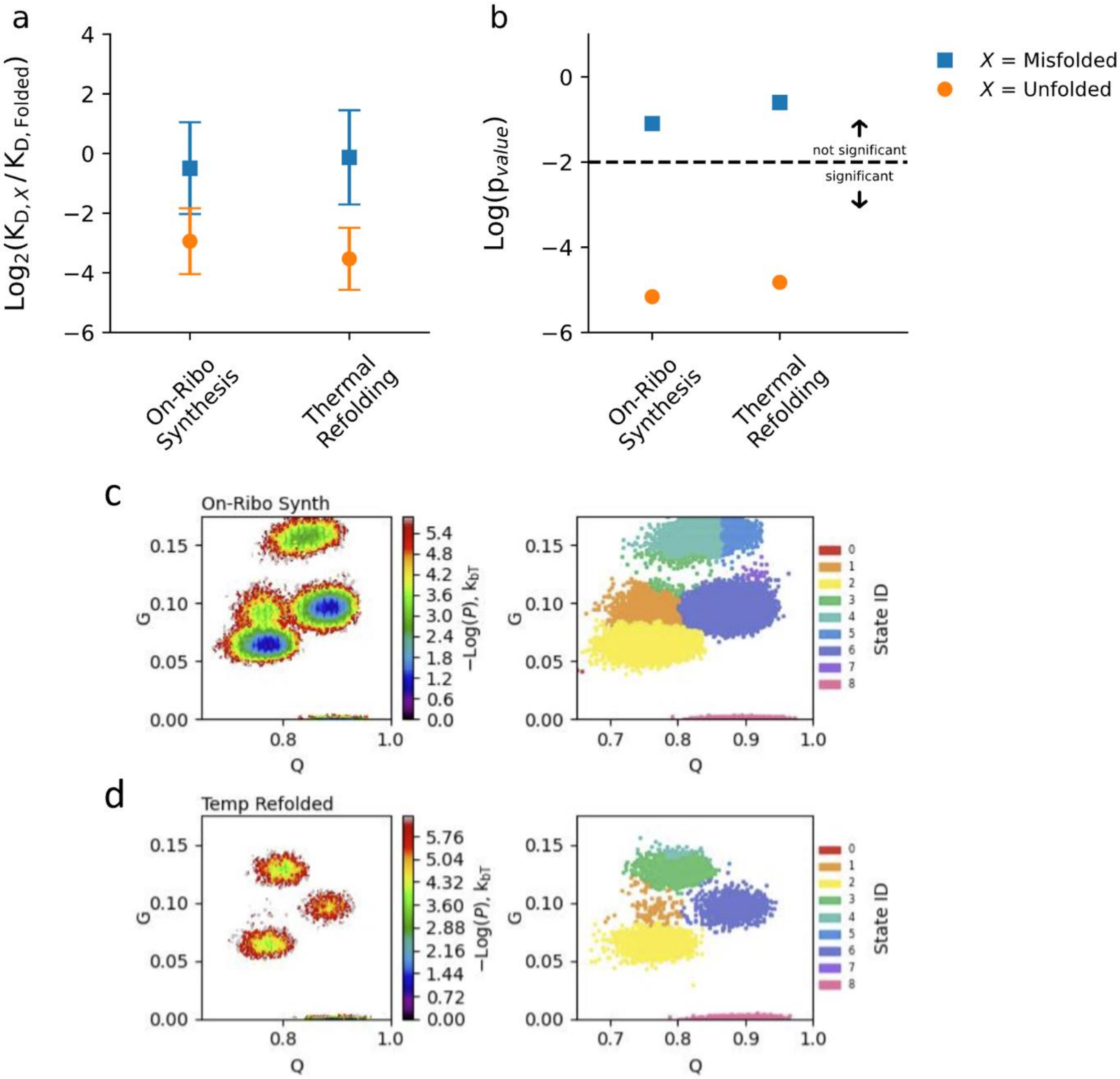

Supplementary Figure 5. Comparison of post-translational and temperature quench results for S-adenosyl methionine synthetase. Comparison between (a) cotranslational folding (On-Ribo Synthesis) and thermal refolding in terms of log K_D for S-adenosyl methionine synthetase. Error bars are 95% confidence intervals about the mean values of 10 independent simulations and (b) log(p_{value}), which indicates the difference between $K_{D,Misfolded}$ and $K_{D,Folded}$ is not statistically significantly, whereas the difference between $K_{D,Unfolded}$ and $K_{D,Folded}$ is statistically significant. p-values were computed using a two-tailed Permutation Test (n=1,000,000). Log probability surface as a function of Q (fraction of native contact) and G (entanglement) for metastable conformational states in the case of (c) on-Ribo synthesis and (d) temperature refolded case for S-adenosylmethionine synthetase.

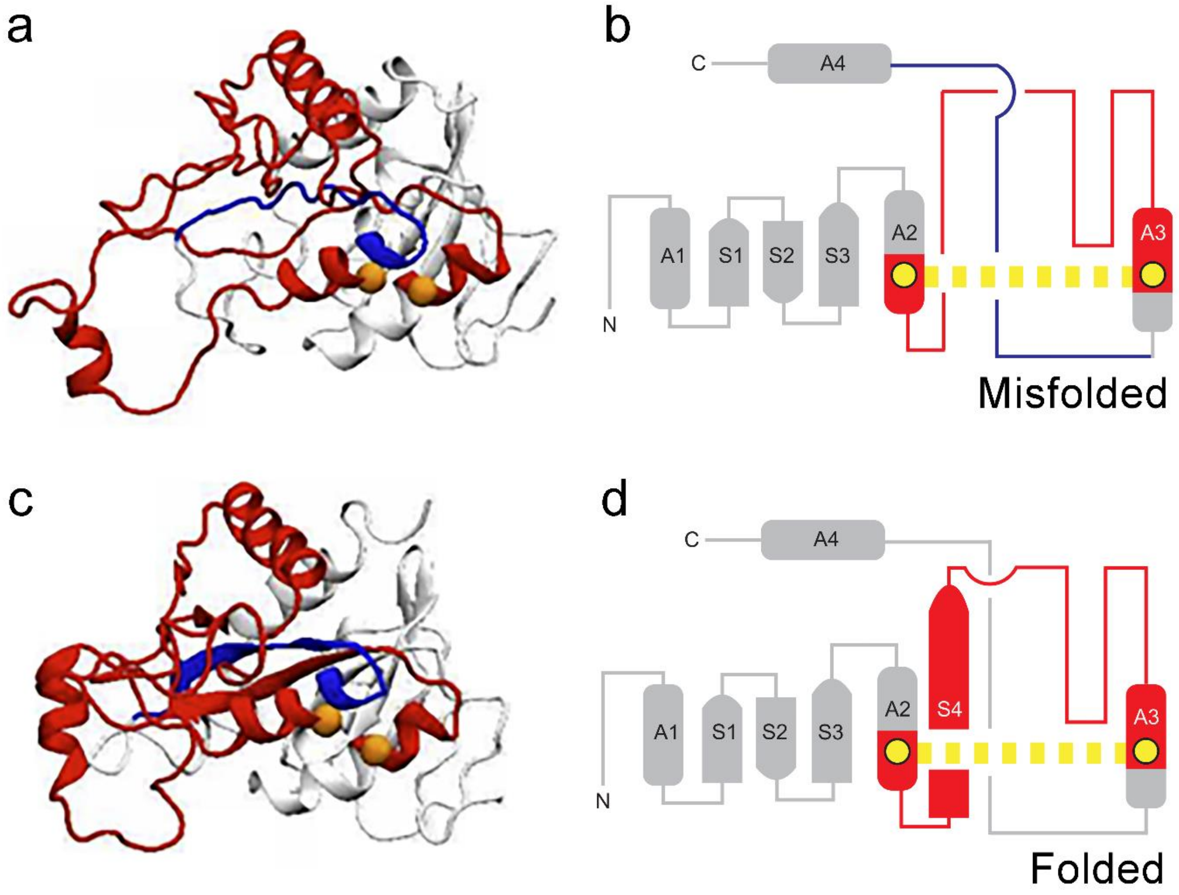

Supplementary Figure 6. Structural representations of near-native entangled (misfolded) and native purine nucleoside phosphorylase. Ribbon representation of the long-lived near-native entangled state and folded state of Purine nucleoside phosphorylase (PDB 1A69). Atomistic structures for all the conformations in all proteins were back mapped here from coarse-grain simulation structures for visualization. The closed loop and threading segment that form the entanglement are colored red and blue, respectively. The pair of residues that form the native contact that closes the loop are highlighted by orange spheres at the location of their C_α atoms.

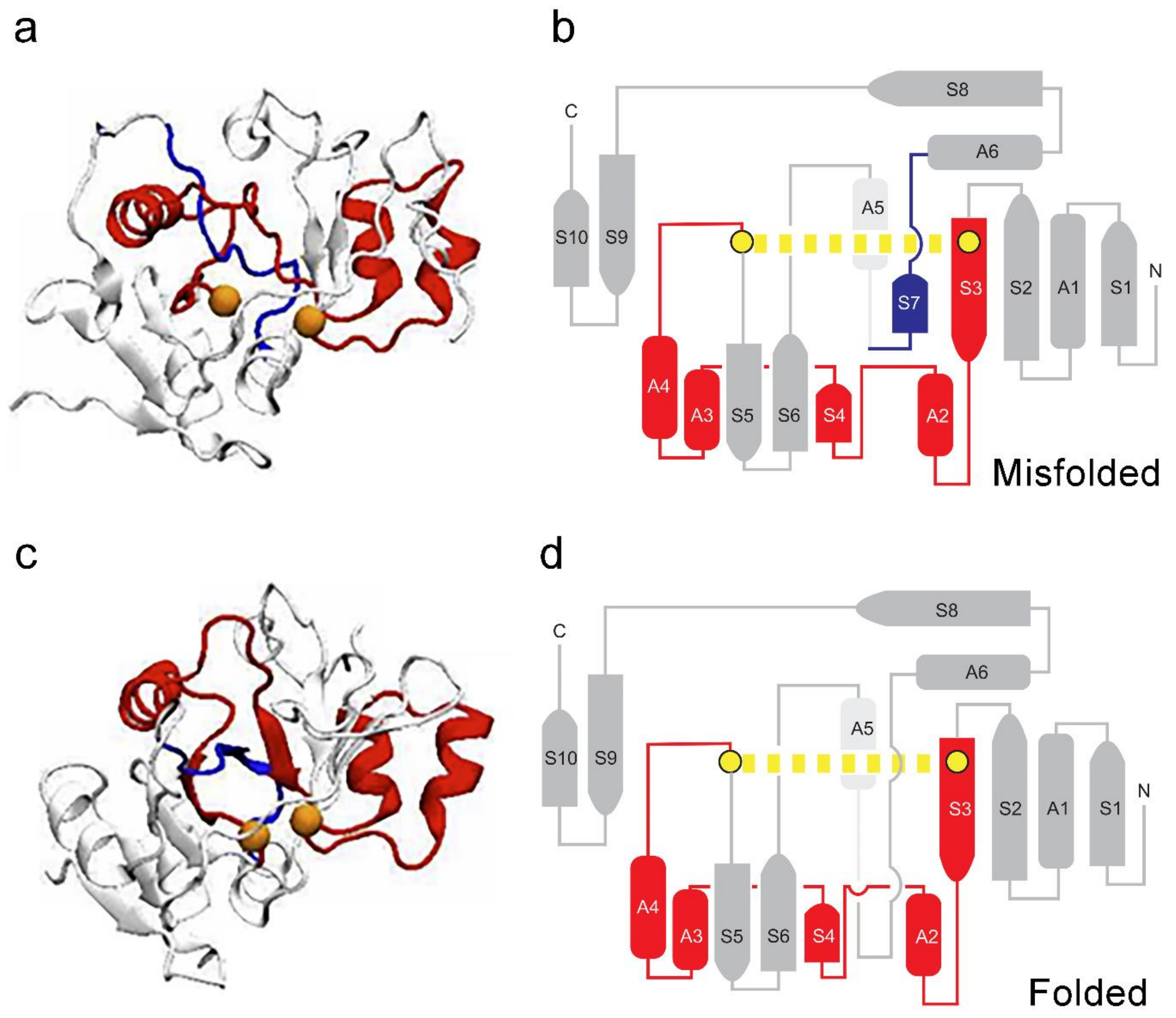

Supplementary Figure 7. Structural representations of near-native entangled (misfolded) and native Transcription Factor 1. Ribbon representation of the long-lived near-native entangled state and folded state of Transcription Factor 1 (PDB 1K7J). Atomistic structures for all the conformations in all proteins were back mapped here from coarse-grain simulation structures for visualization. The closed loop and threading segment that form the entanglement are colored red and blue, respectively. The pair of residues that form the native contact that closes the loop are highlighted by orange spheres at the location of their C_α atoms.

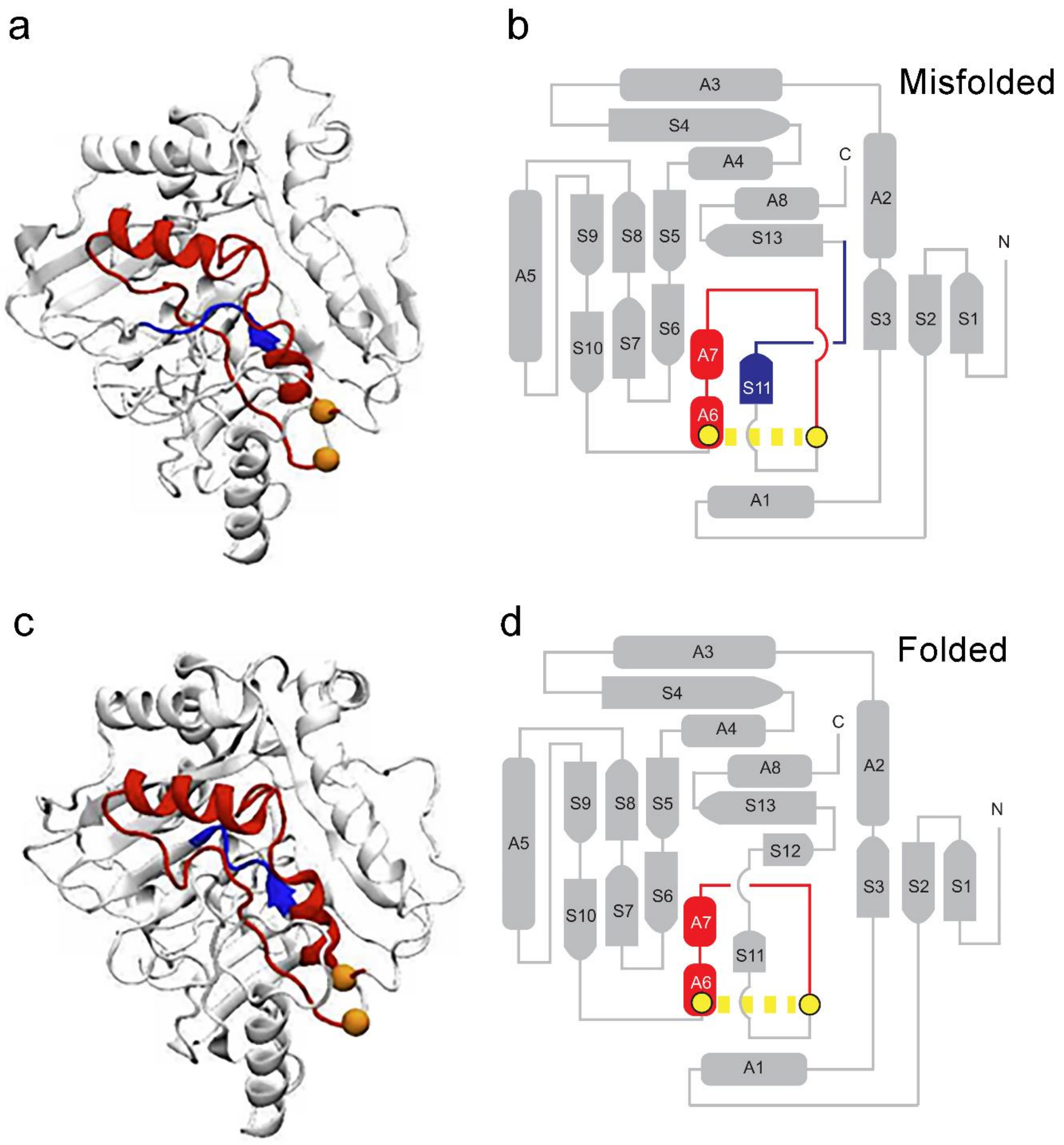

Supplementary Figure 8. Structural representations of near-native entangled (misfolded) and native isochorismate synthase. Ribbon representation of the long-lived near-native entangled state and folded state of isochorismate synthase (PDB 3HWO). Atomistic structures for all the conformations in all proteins were back mapped here from coarse-grain simulation structures for visualization. The closed loop and threading segment that form the entanglement are colored red and blue, respectively. The pair of residues that form the native contact that closes the loop are highlighted by orange spheres at the location of their C_α atoms.

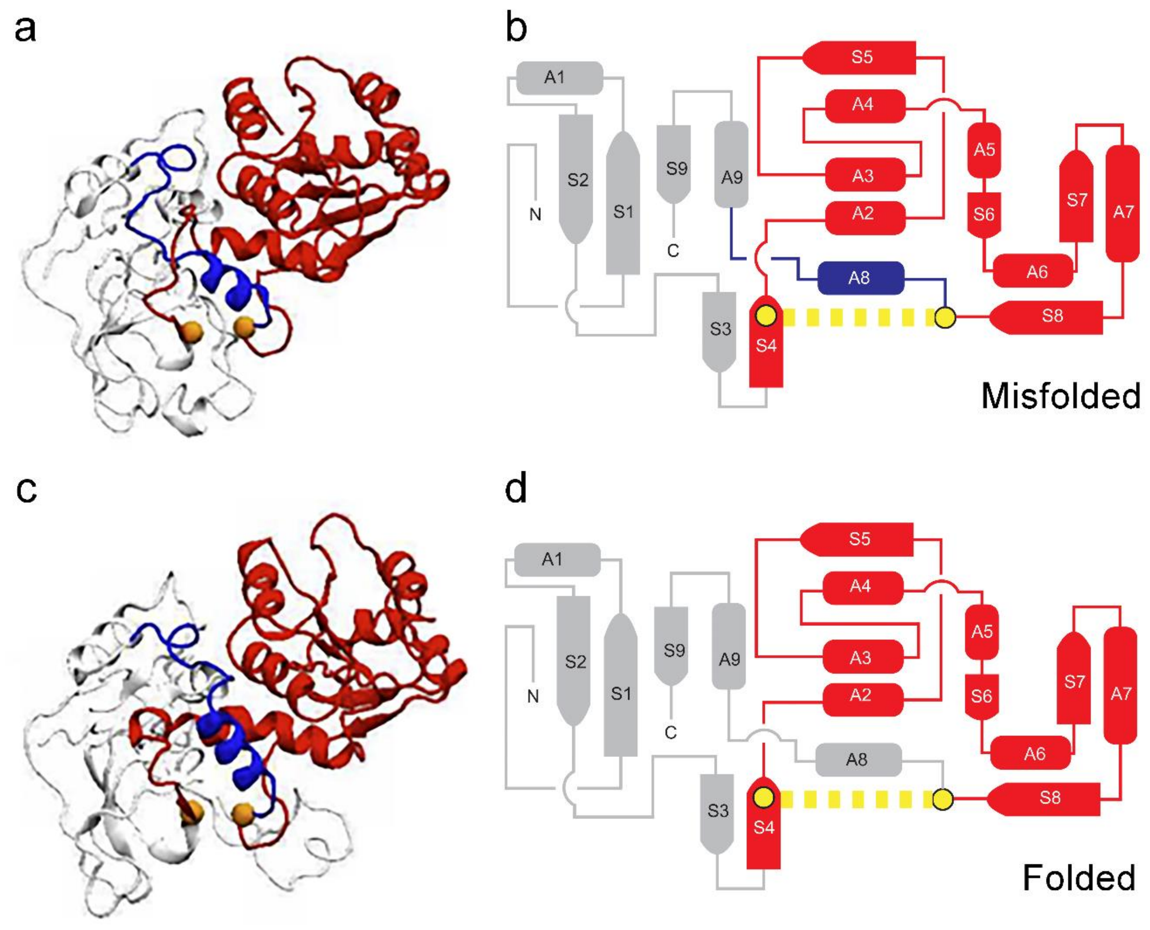

Supplementary Figure 9. Structural representations of near-native entangled (misfolded) and native galactitiol-1-phosphate dehydrogenase. Ribbon representation of the long-lived near-native entangled state and folded state of Galactitiol-1-phosphate dehydrogenase (PDB 4A2C). Atomistic structures for all the conformations in all proteins were back mapped here from coarse-grain simulation structures for visualization. The closed loop and threading segment that form the entanglement are colored red and blue, respectively. The pair of residues that form the native contact that closes the loop are highlighted by orange spheres at the location of their C_α atoms.

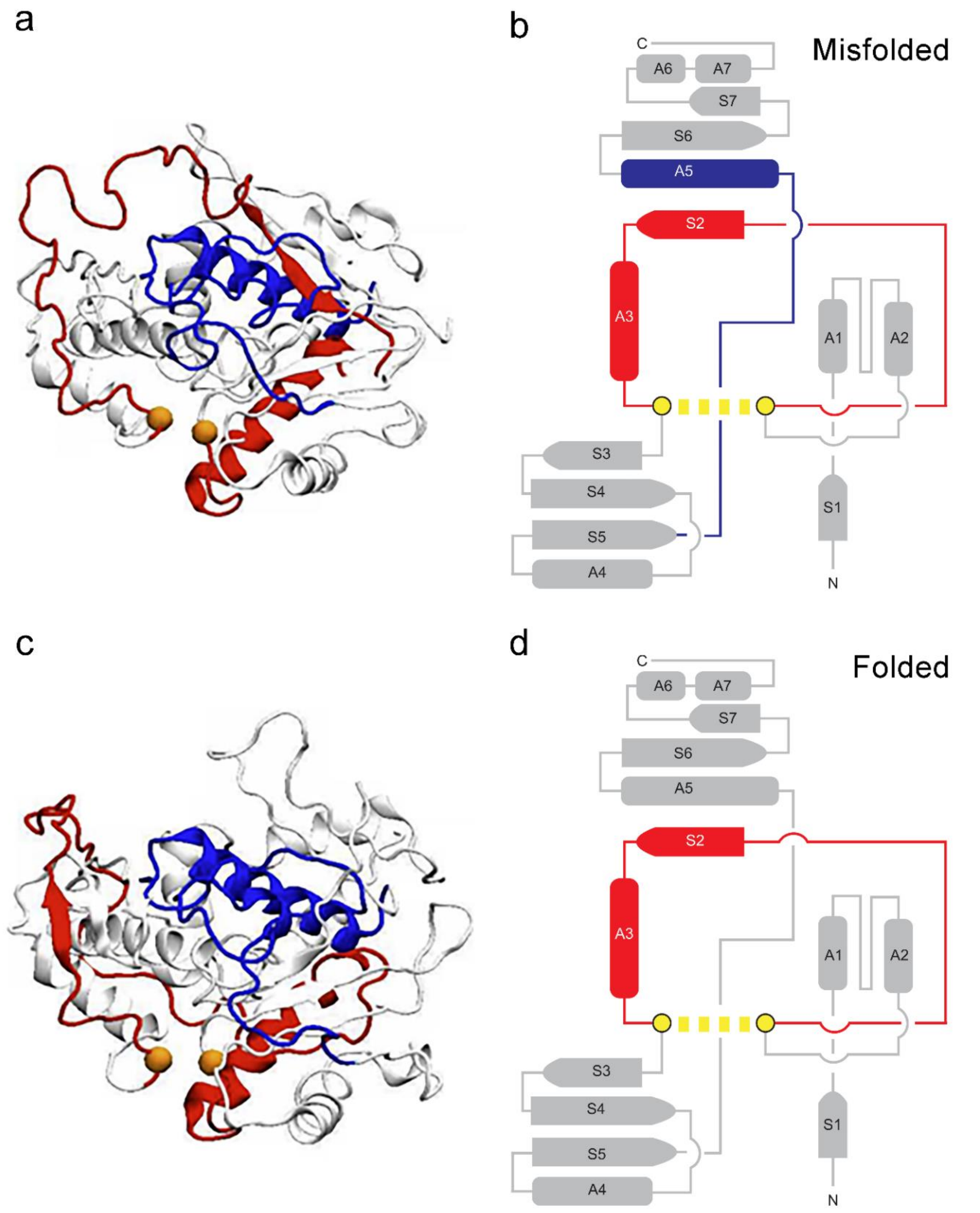

Supplementary Figure 10. Structural representations of near-native entangled (misfolded) and native S-adenosylmethionine synthetase. Ribbon representation of the long-lived near-native entangled state and folded state of S-adenosylmethionine synthetase (PDB 1P7L). Atomistic structures for all the conformations in all proteins were back mapped here from coarse-grain simulation structures for visualization. The closed loop and threading segment that form the entanglement are colored red and blue, respectively. The pair of residues that form the native contact that closes the loop are highlighted by orange spheres at the location of their C_α atoms.

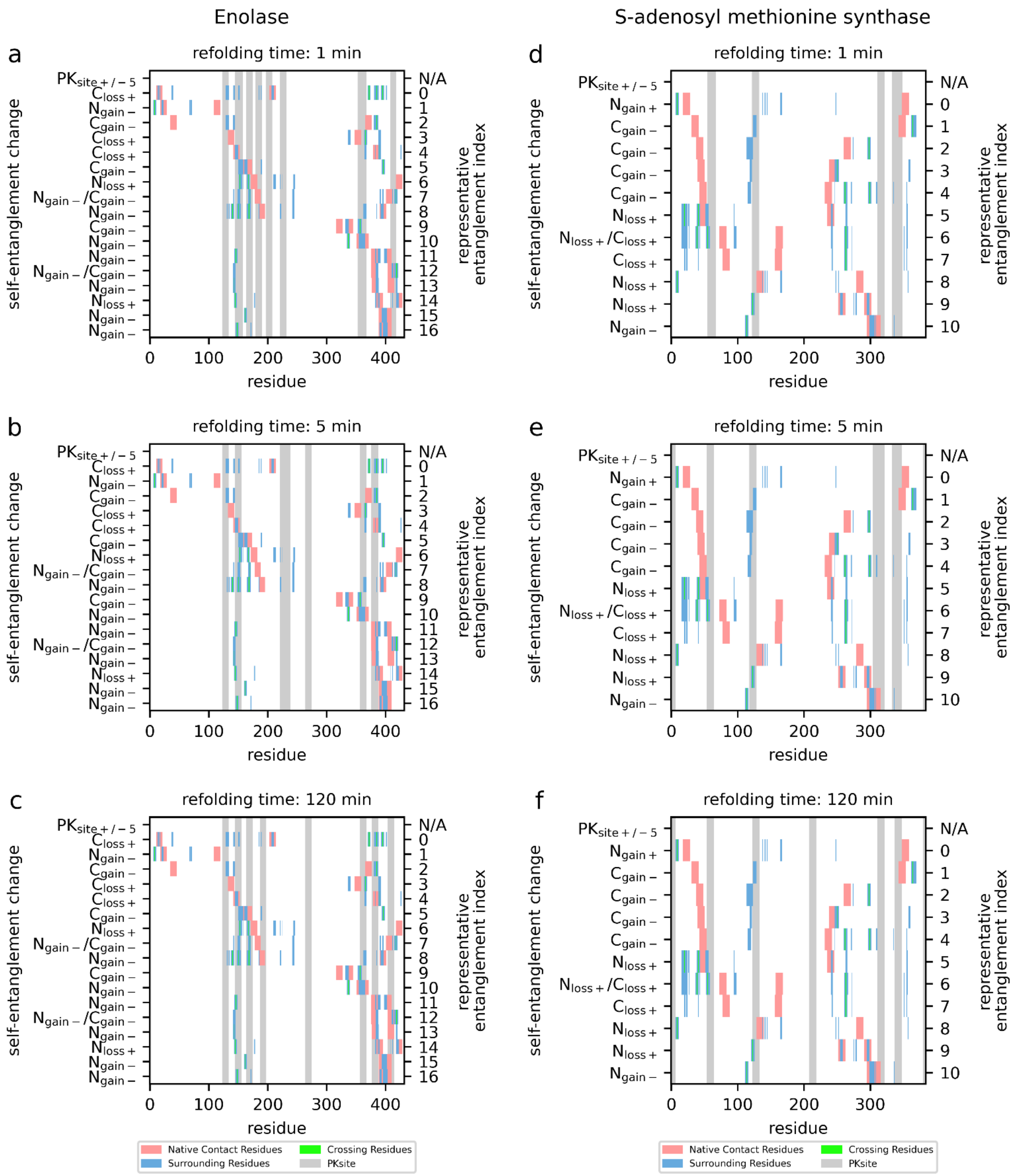

Supplementary Figure 11. Representative changes in entanglement after clustering. Results for 2FYM (left) and 1P7L (right) organized by the start of the loop (pink). The crossing residues determined by Topoly are shown in (green) and the residues within 8A of those crossings or within 5 residues on the native contact are shown in (blue). The PK cut sites and the 5 residues surrounding them are shown in (grey). The left axis describes the change type observed for this representative change in self-entanglement. For example N_{gain+} signifies a loop that was not threaded in the native state is threaded in the misfolded state with a positive chirality. The right axis gives the representative entanglement index from Supplementary Table 11 and

12. The three rows correspond to the three LiP-MS time points (1 min top, 5 min middle, 120 min, bottom).

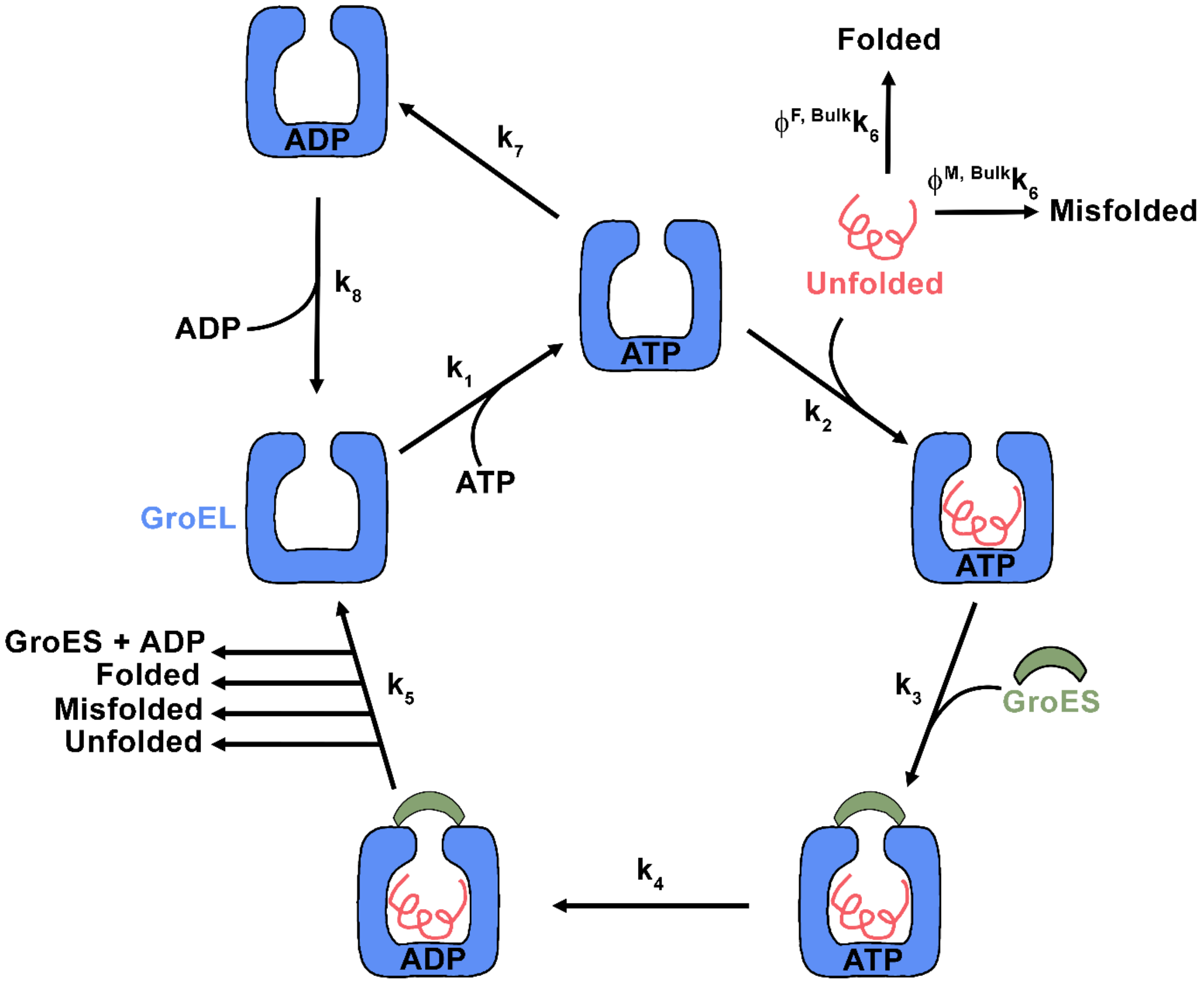

Supplementary Figure 12. Kinetic reaction scheme followed by Eq. 7. All parameters are as described in the Methods.

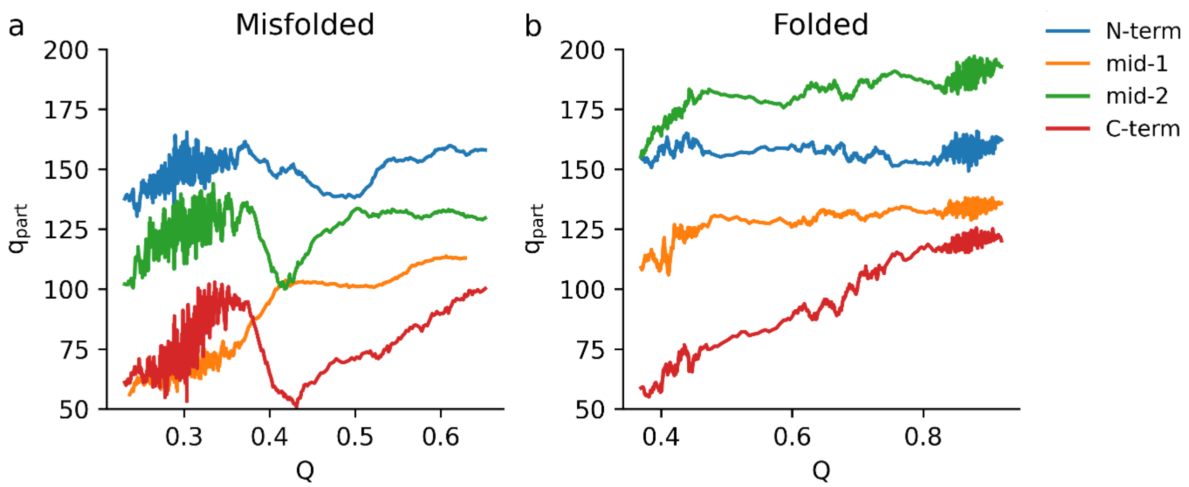

Supplementary Figure 13. The number of native contacts versus Q indicates backtracking is required to disentangle misfolded states of isochorismate synthase. Results are shown for trajectories that misfold (a) and fold (b). The variation of total number of contacts of four different parts (q_{part}) of the protein is plotted as a function of fraction of native contacts (Q). N-term corresponds to residues 1-100; mid-1 is residues 101-200; mid-2 is residues 201-300; C-term is residues 301-391.

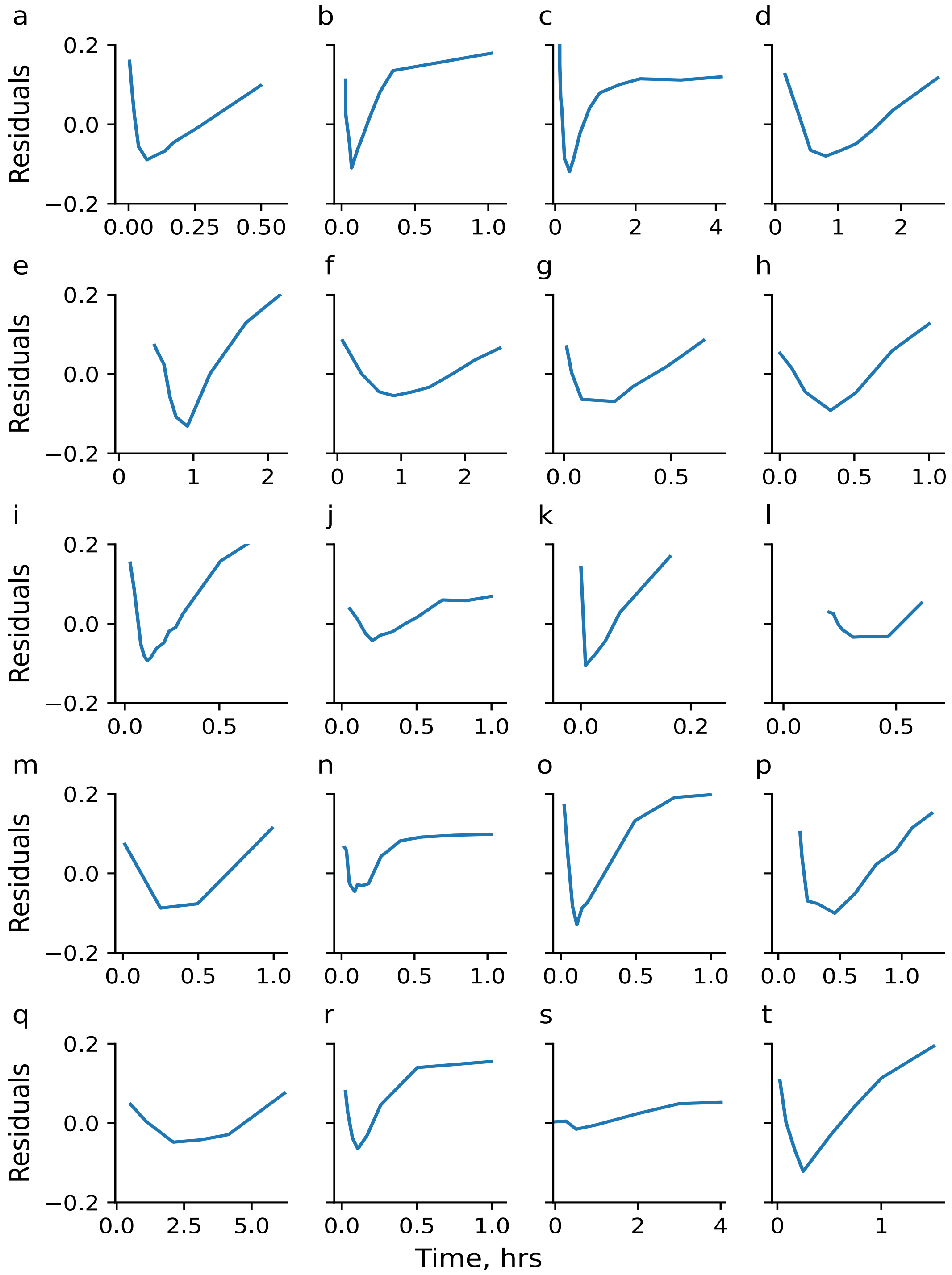

Supplementary Figure 14. Residuals from fits to 20 experimental refolding studies with single-exponential kinetic fit. The residuals, computed as (experimental value) – (fit value), are shown for each of the 20 experimental studies.

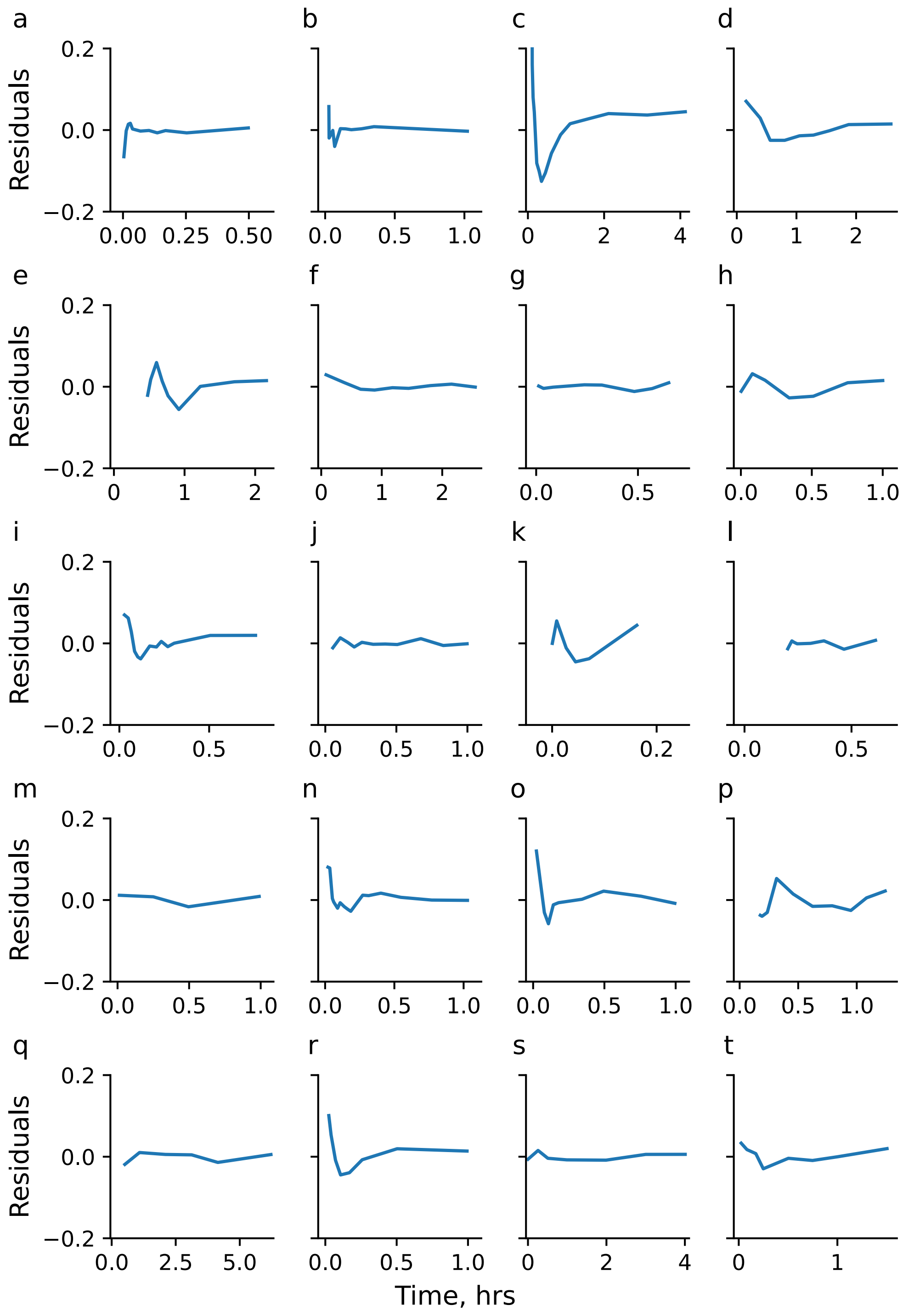

Supplementary Figure 15. Residuals from fits to 20 experimental refolding studies with double-exponential kinetic fit. The residuals, computed as (experimental value) – (fit value), are shown for each of the 20 experimental studies.

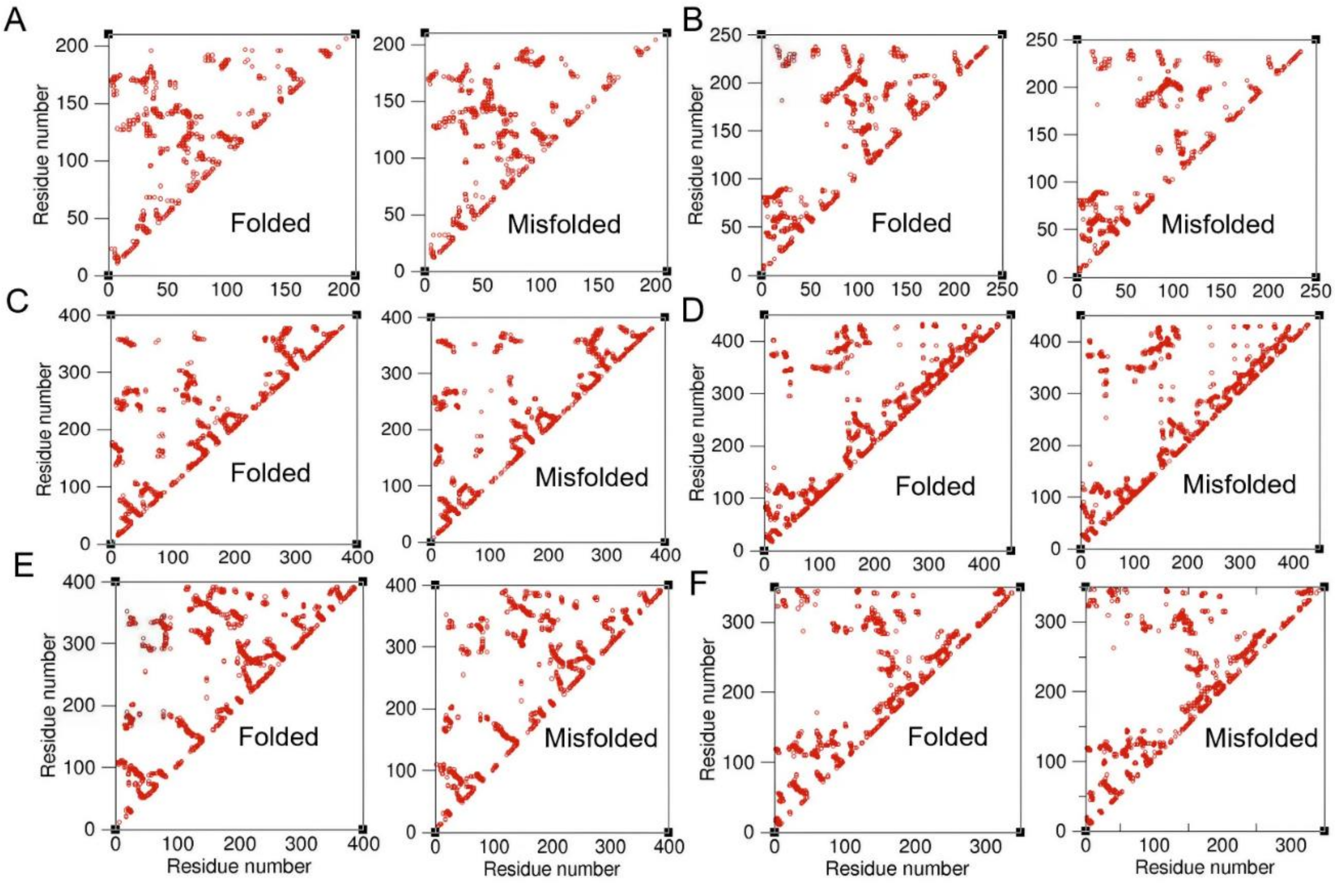

Supplementary Figure 16. Contact map analysis of folded and misfolded states of all the six-client proteins studied here. (A) Transcription factor 1, (B) Purine nucleoside phosphorylase, (C) S-adenosyl methionine synthase, (D) Enolase, (E) Isochorismate synthase, (F) Galactitiol-1-phosphate dehydrogenase.

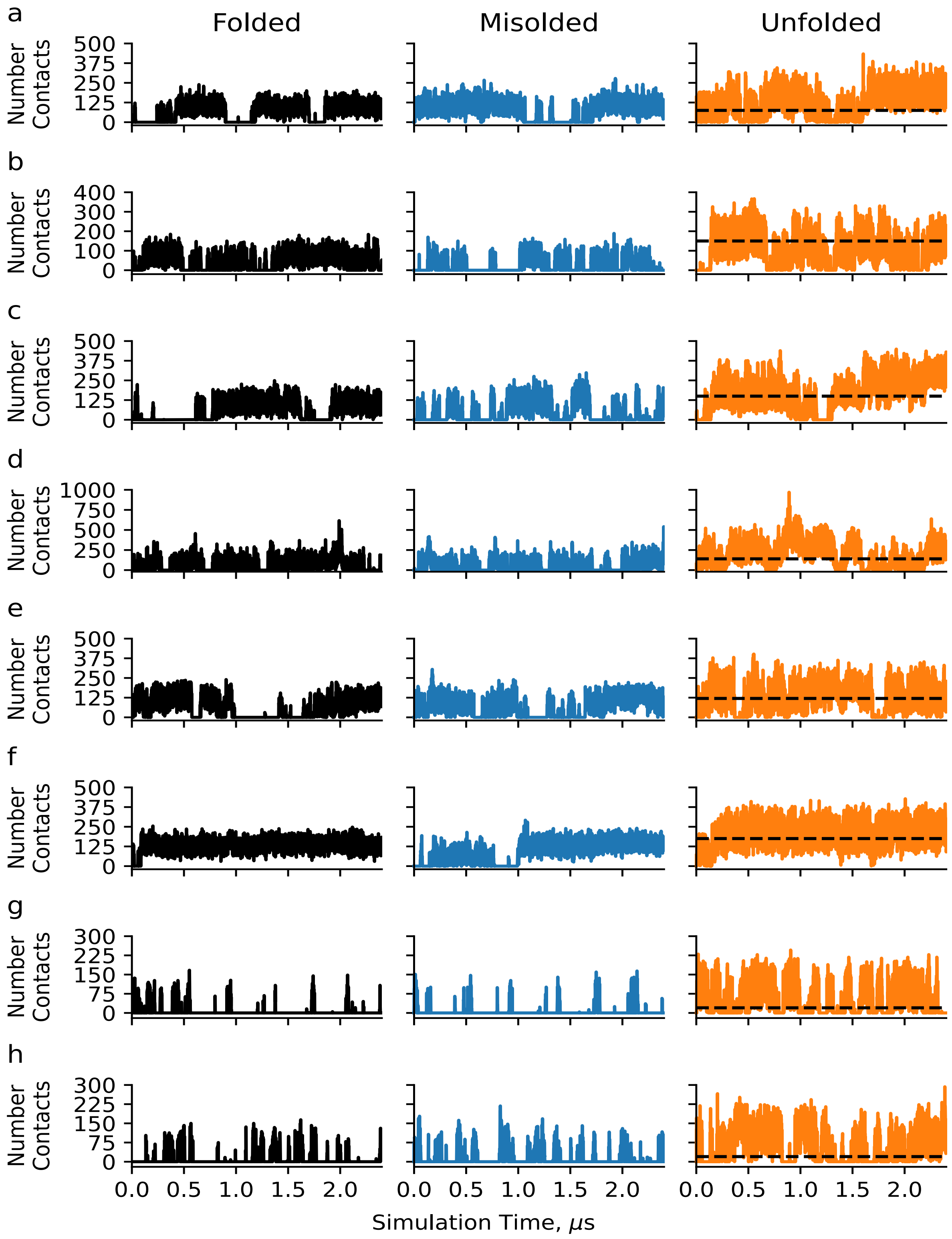

Supplementary Figure 17. Variation of total number of contacts between unfolded, folded and misfolded client protein with the chaperone GroEL, HtpG and DnaK. (a) GroEL-Purine nucleoside phosphorylase, (b) GroEL-Transcription factor 1, (c) GroEL-S-adenosylmethionine synthetase, (d) GroEL-Enolase, (e) GroEL-Isochorismate synthase, (f) GroEL-Galactitol-1-phosphate dehydrogenase, (g) HtpG-

Purine nucleoside phosphorylase and (h) DnaK-Purine nucleoside phosphorylase. Contact thresholds for each system are shown by the black dotted horizontal lines.
