# Supplementary material for: How soluble misfolded proteins bypass chaperones at the molecular level
Source: Nat Commun. 2023 Jun 21;14:3689. doi: 10.1038/s41467-023-38962-z (PMC10284856; doi:10.1038/s41467-023-38962-z)
Supplement: Supplementary file 2 — Description to Additional Supplementary Information [file 41467_2023_38962_MOESM2_ESM.pdf]

Description of Additional Supplementary Files

File Name: Supplementary Dataset 1

Description: This data file indicates our meta-analyses study based on twenty experimental papers about chaperone induced refolding of protein. This file includes important details about those experimental studies.

File Name: Supplementary Dataset 2

Description: Experimental results of chaperone-protein binding K_D values. We compared these experimental K_D values with our calculated K_D values.

File Name: Supplementary Movie 1

Description: Sample trajectories of all the chaperone-client protein systems studied in this work. These trajectories show the binding simulation of the six-client protein (Isochorismate synthase, Enolase, Galactitol-1-phosphate dehydrogenase, Transcription factor 1, S-adenosylmethionine synthetase and Purine nucleoside phosphorylase) in folded, misfolded and unfolded states with the chaperone (GroEL or HtpG or DnaK).
