# Supplementary material for: How soluble misfolded proteins bypass chaperones at the molecular level
Source: Nat Commun. 2023 Jun 21;14:3689. doi: 10.1038/s41467-023-38962-z (PMC10284856; doi:10.1038/s41467-023-38962-z)
Supplement: Supplementary file 6 — Reporting Summary [file 41467_2023_38962_MOESM6_ESM.pdf]

nature portfolio

Corresponding author(s): Edward P. O'Brien
Last updated by author(s): 2023/04/24

Reporting Summary

Nature Portfolio wishes to improve the reproducibility of the work that we publish. This form provides structure for consistency and transparency in reporting. For further information on Nature Portfolio policies, see our Editorial Policies and the Editorial Policy Checklist.

Statistics

For all statistical analyses, confirm that the following items are present in the figure legend, table legend, main text, or Methods section.

n/a	Confirmed	
☐	☒	The exact sample size (*n*) for each experimental group/condition, given as a discrete number and unit of measurement
☐	☒	A statement on whether measurements were taken from distinct samples or whether the same sample was measured repeatedly
☐	☒	The statistical test(s) used AND whether they are one- or two-sided<br>*Only common tests should be described solely by name; describe more complex techniques in the Methods section.*
☒	☐	A description of all covariates tested
☒	☐	A description of any assumptions or corrections, such as tests of normality and adjustment for multiple comparisons
☐	☒	A full description of the statistical parameters including central tendency (e.g. means) or other basic estimates (e.g. regression coefficient) AND variation (e.g. standard deviation) or associated estimates of uncertainty (e.g. confidence intervals)
☐	☒	For null hypothesis testing, the test statistic (e.g. *F*, *t*, *r*) with confidence intervals, effect sizes, degrees of freedom and *P* value noted<br>*Give P values as exact values whenever suitable.*
☒	☐	For Bayesian analysis, information on the choice of priors and Markov chain Monte Carlo settings
☒	☐	For hierarchical and complex designs, identification of the appropriate level for tests and full reporting of outcomes
☐	☒	Estimates of effect sizes (e.g. Cohen's *d*, Pearson's *r*), indicating how they were calculated

Our web collection on statistics for biologists contains articles on many of the points above.

Software and code

Policy information about availability of computer code

Data collection	Simulation were run in CHARMM v35 and OpenMM v7.4.1. Analysis were carred out using Webplotdigitizer (version 4.4), visual molecular dynamics (v 1.9.1) and Pyhton 3.6. The OpenMM input files, Python (including the SciPy v 1.7.3) and Fortran (v f.90)and CHARMM scripts, and Visual Molecular Dynamics v1.9.1 analysis codes, sample commands, and example outputs are available in the GitHub under accession code https://github.com/obrien-lab-psu/. We provided all other scripts, input files and codes for the analysis in this study at https://github.com/obrien-lab-psu/. Software used in experimental data analysis is available in the GitHub under the accession code https://github.com/FriedLabJHU/Refoldability-Tools.
Data analysis	Analysis was carried out using custom python code.

For manuscripts utilizing custom algorithms or software that are central to the research but not yet described in published literature, software must be made available to editors and reviewers. We strongly encourage code deposition in a community repository (e.g. GitHub). See the Nature Portfolio guidelines for submitting code & software for further information.

Data

Policy information about availability of data

All manuscripts must include a data availability statement. This statement should provide the following information, where applicable:

- Accession codes, unique identifiers, or web links for publicly available datasets
- A description of any restrictions on data availability
- For clinical datasets or third party data, please ensure that the statement adheres to our policy

The data from mass spectrometry proteomics was generated in this study and have been deposited to the ProteomeXchange Consortium via the PRIDE partner repository with the data set identifier PXD030869 https://www.ebi.ac.uk/pride/archive/projects/PXD030869. Summary data for these experiments are also provided in Supplementary Table 10, 11, 12, 13 and Supplementary Figure 11. The processed data generated in this study are provided in the Source Data file and sample simulation trajectories of the systems studied here have been shown in the Supplementary Movie 1. Source data are provided with this paper.

Research involving human participants, their data, or biological material

Policy information about studies with human participants or human data. See also policy information about sex, gender (identity/presentation), and sexual orientation and race, ethnicity and racism.

Reporting on sex and gender	Not applicable.
Reporting on race, ethnicity, or other socially relevant groupings	Not applicable.
Population characteristics	Not applicable.
Recruitment	Not applicable.
Ethics oversight	Not applicable.

Note that full information on the approval of the study protocol must also be provided in the manuscript.

Field-specific reporting

Please select the one below that is the best fit for your research. If you are not sure, read the appropriate sections before making your selection.

☒ Life sciences ☐ Behavioural & social sciences ☐ Ecological, evolutionary & environmental sciences

For a reference copy of the document with all sections, see nature.com/documents/nr-reporting-summary-flat.pdf

Life sciences study design

All studies must disclose on these points even when the disclosure is negative.

Sample size	Precisely 8 chaperone-client protein systems with 3 client protein conformations and each of which have 10 independent trajectories. Total 240 simulations were performed. 10 trajectories per system was chosen so that we can get good enough statistics with robust sampling and for each trajectory, we performed 2.4 microsecond long simulation (Total 576 microsecond of simulation for all 240 systems). The simulation statistics and time scales are good enough that lead to multiple binding/unbinding event between chaperone and client protein as evidenced from their trajectories.
Data exclusions	No data excluded.
Replication	Each binding simulation was replicated 10 times (per protein conformation).
Randomization	No experimental groups or randomization was carried out.
Blinding	Blinding is not relevant here. We performed molecular dynamics simulations and analyzed the results. We did not perform any clinical trials. There is no drug related or medicinal studies performed here, hence blinding is out of scope from the current study.

Reporting for specific materials, systems and methods

We require information from authors about some types of materials, experimental systems and methods used in many studies. Here, indicate whether each material, system or method listed is relevant to your study. If you are not sure if a list item applies to your research, read the appropriate section before selecting a response.

Materials & experimental systems

n/a	Involved in the study
☒	☐ Antibodies
☒	☐ Eukaryotic cell lines
☒	☐ Palaeontology and archaeology
☒	☐ Animals and other organisms
☒	☐ Clinical data
☒	☐ Dual use research of concern
☒	☐ Plants

Methods

n/a	Involved in the study
☒	☐ ChIP-seq
☒	☐ Flow cytometry
☒	☐ MRI-based neuroimaging
