# Supplementary material for: How soluble misfolded proteins bypass chaperones at the molecular level
Source: Nat Commun. 2023 Jun 21;14:3689. doi: 10.1038/s41467-023-38962-z (PMC10284856; doi:10.1038/s41467-023-38962-z)
Supplement: Supplementary file 7 — Source Data [file 41467_2023_38962_MOESM7_ESM.zip › Table of Content of Source data file.docx]

**Table of Content of Source data file**

| **Figure number** | **‘Folder name’ where the Data/Script is available in the ‘Source Data’ file** |
| --- | --- |
| Figure 1 | Source Data/meta_analysis_data/check.py |
| Figure 2 | Source Data/meta_analysis_data/5.dat |
| Figure 3 | Source Data/ calc_native_contact_fraction_v2 |
| Figure 4 | Source Data/ molecular_dynamics/input_files_and_scripts  /binding_simlation_analysis_code/  Source Data/ molecular_dynamics/input_files_and_scripts  /kd/ |
| Figure 5 | Source Data/permutation_test_for_model_consistency_with_experimental_data  /codes/backmap_pulchra_only.py |
| Figure 6 | Source Data/Folded and Misfolded structures/ |

| **Table number** | **‘Folder name’ where the Data/Script is available in the ‘Source Data’ file** |
| --- | --- |
| Table 1 | Source Data/meta_analysis_data/check.py |
| Table 2 | Source Data/ molecular_dynamics/input_files_and_scripts  /binding_simlation_analysis_code/ |
| Table 3 | Source Data/ calc_native_contact_fraction_v2  Source Data/permutation_test_for_model_consistency_with_experimental_data  /codes/ |

| **Figure number** | **‘Folder name’ where the Data/Script is available in the ‘Source Data’ file** |
| --- | --- |
| Supplementary Figure 1 | Source Data/meta_analysis_data/check.py |
| Supplementary Figure 2 | Source Data/atp_consumption  Source Data/ molecular_dynamics/input_files_and_scripts  /atp_consump |
| Supplementary Figure 3 | Source Data/q_rg/q_rg.agr |
| Supplementary Figure 4 | Source Data/ molecular_dynamics/diff-cont-cutoff  Source Data/ molecular_dynamics/input_files_and_scripts  /binding_simlation_analysis_code |
| Supplementary Figure 5 | Source Data/ molecular_dynamics/input_files_and_scripts  /kd/  Source Data/ q_rg/each_u_f_m_f_permutation.py |
| Supplementary Figure 6 | Source Data/Folded and Misfolded structures/fold_1a69_rebuilt  Source Data/Folded and Misfolded structures/misf_1a69_rebuilt |
| Supplementary Figure 7 | Source Data/Folded and Misfolded structures/ fold_1k7j_rebuilt  Source Data/Folded and Misfolded structures/misf_1k7j_rebuilt |
| Supplementary Figure 8 | Source Data/Folded and Misfolded structures/fold_3hwo_rebuilt  Source Data/Folded and Misfolded structures/misf_3hwo_rebuilt |
| Supplementary Figure 9 | Source Data/Folded and Misfolded structures/fold_4a2c_rebuilt  Source Data/Folded and Misfolded structures/misf_4a2c_rebuilt |
| Supplementary Figure 10 | Source Data/Folded and Misfolded structures/fold_1p7l_rebuilt  Source Data/Folded and Misfolded structures/misf_1p7l_rebuilt |
| Supplementary Figure 11 | Source Data/permutation_test_for_model_consistency_with  _experimental_data/ |
| Supplementary Figure 13 | Source Data/ molecular_dynamics/backtrack_data |
| Supplementary Figure 14 | Source Data/meta_analysis_data/residual/single_exp_res |
| Supplementary Figure 15 | Source Data/meta_analysis_data/residual/double_exp_res |
| Supplementary Figure 16 | Source Data/ molecular_dynamics/cont_map/aa |
| Supplementary Figure 17 | Source Data/ molecular_dynamics/total_no_contact/  Source Data/ molecular_dynamics/input_files_and_scripts  /binding_simlation_analysis_code |

| **Table number** | **‘Folder name’ where the Data/Script is available in the ‘Source Data’ file** |
| --- | --- |
| Supplementary Table 1 | Source Data/meta_analysis_data |
| Supplementary Table 2 | Source Data/ molecular_dynamics/input_files_and_scripts  /atp_consump |
| Supplementary Table 3 | Source Data/ molecular_dynamics/input_files_and_scripts  /atp_consump |
| Supplementary Table 5 | Source Data/ molecular_dynamics/input_files_and_scripts  /kd |
| Supplementary Table 6 | Source Data/q_rg/each_u_f_m_f_permutation.py |
| Supplementary Table 7 | Source Data/ molecular_dynamics/input_files_and_scripts  /binding_simlation_analysis_code |
| Supplementary Table 10 | Source Data/permutation_test_for_model_consistency_with_experimenta  l_data |
| Supplementary Table 11 | Source Data/permutation_test_for_model_consistency_with_experimenta  l_data |
| Supplementary Table 12 | Source Data/permutation_test_for_model_consistency_with_experimenta  l_data |
| Supplementary Table 13 | Source Data/permutation_test_for_model_consistency_with_experimenta  l_data |
| Supplementary Table 17 | Source Data/permutation_test_for_model_consistency_with_experimental_data  /codes/entanglement_analysis_v2.0.py |
